# Neuromorphic Computing Using Synaptic Plasticity of Supercapacitors

**DOI:** 10.1002/advs.202500521

**Published:** 2025-03-24

**Authors:** Ling Wang, Xing Liu, Guangcai Zhang, Fuxun Qi, Xi Chen

**Affiliations:** ^1^ School of Artificial Intelligence Science and Technology University of Shanghai for Science and Technology Shanghai 200093 China; ^2^ Institute of Photonic Chips University of Shanghai for Science and Technology Shanghai 200093 China; ^3^ School of Materials and Chemistry University of Shanghai for Science and Technology Shanghai 200093 China

**Keywords:** artificial neural network, braille recognition, diffractive deep neural network, neuromorphic computing, plasticity, supercapacitor

## Abstract

Neuromorphic computing systems convert multimodal signals to electrical responses for artificial intelligence recognition. Energy is consumed during both the response enhancement and depression, making the systems suffer from high energy consumption. This study presents a neuromorphic computing pathway based on supercapacitors. MXene Ti₃C₂Tx supercapacitors are fabricated and convert current stimuli to voltage responses. The response enhancement and depression are tunable through adjusting charging and discharging current stimuli, thus exhibiting synaptic plasticity. Typical synaptic behaviors are demonstrated, including short‐term memory, long‐term memory, paired‐pulse facilitation, and learning experience. Next, the voltage responses are used to recognize Braille numbers represented by 3 × 4 arrays. A charging/discharging current pulse train representing each Braille array is applied to the supercapacitor. The voltage responses are collected and converted to 12‐pixel greyscale images. Once the images representing Braille numbers 0–9 are input into artificial neural networks and deep diffraction neural networks, 100% accuracy can be achieved for recognizing the ten numbers. Because energy is stored during response enhancement in the supercapacitor and released once the response declines, this research demonstrates the potential applications of energy storage devices in neuromorphic computing, providing an innovative way to develop energy‐efficient brain‐like computing systems.

## Graphical abstract

A neuromorphic computing pathway based on supercapacitors is presented. MXene Ti₃C₂Tx supercapacitors demonstrate voltage responses under current stimuli, thus exhibiting synaptic plasticity. Energy is stored during response enhancement and released once the response declines. The voltage responses are used to recognize Braille numbers 0–9 assisted by an artificial neural network and a deep diffraction neural network. 100% accuracy is achieved.

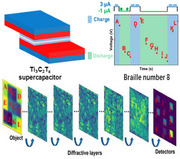

## 1 Introduction

Traditional von Neumann architecture separates storage and computational units, which results in frequent data transfers.^[^ ^1^ ^]^ This consumes substantial energy and creates performance bottlenecks, particularly in large‐scale data processing and artificial intelligence tasks.^[^ ^2^ ^]^ On the other hand, neuromorphic computing integrates computation and storage into the same hardware unit, reduces the need for data transfers, and provides a novel pathway for efficient computation architectures.^[^ ^3^ ^]^ In neuromorphic computing systems, multimodal inputs, such as optical, mechanical, and chemical, are converted into electrical signals. The electrical signals are processed by artificial neural networks (ANN) for recognition through weight updates. However, current multimodal signal conversion technologies face challenges in high energy consumption.^[^ ^4^ ^]^ Artificial synapses, mimicking a central role in signal transmission between neurons, have become a focus for signal conversion in neuromorphic computing.^[^ ^5^ ^]^ Enhancement and depression of synapse responses depend on forward and reverse signal stimulation. The property that responses are tunable under different signal parameters, so‐called synaptic plasticity, is fundamental for neuromorphic computing.^[^ ^6^ ^]^ However, energy is consumed no matter whether the responses enhance or depress, making synapses unsuitable for low‐energy response generation.^[^ ^7^ ^]^ There is an urgent need for neuromorphic computing systems to develop an innovative paradigm of signal‐response conversion.^[^ ^8^ ^]^

Supercapacitors, with their unique current‐voltage response characteristics, show great potential in energy storage systems.^[^ ^9^ ^]^ They offer higher power density and longer operational lifespans than traditional batteries. The state of charge (SoC) and state of discharge (SoD) of supercapacitors are realized through charging and discharging current stimulation, respectively.^[^ ^10^ ^]^ The phenomenon is that the supercapacitor voltages are tunable under different charge/discharge currents similar to synaptic plasticity and have great potential for neuromorphic computing.^[^ ^11^ ^]^ In this case, energy is stored during response enhancement and is released once the response declines. Recently, scientists have involved energy storage devices to achieve weight updates in neuromorphic computing.^[^ ^12^ ^]^ However, the utilization of supercapacitors for signal conversion in neuromorphic computing has not been investigated. By incorporating supercapacitors into neuromorphic computing systems, signal conversion can be realized without significantly increasing energy consumption, offering a new pathway for efficient computing architectures.^[^ ^13^ ^]^

This study presents a neuromorphic computing pathway in which signal collection is based on the current stimulation of MXene Ti₃C₂T_x_ supercapacitors.^[^ ^14^ ^]^ The supercapacitor voltages are controlled by adjusting charging/discharging currents and durations. Due to the plasticity, typical synaptic behaviors are demonstrated, including short‐term memory (STM), long‐term memory (LTM), paired‐pulse facilitation (PPF), and learning experience. Current pulse trains are encoded by Braville numbers and applied to the Mxene supercapacitor. Voltage responses are collected as the input of artificial neural networks (ANN) and deep diffraction neural networks (D^2^NN). High accuracies are achieved for classifying Braille numbers 0–9.

## 2 Results and Discussion

### 2.1 Mechanism of Supercapacitor‐Based Neuromorphic Computing

**Figure 1:**
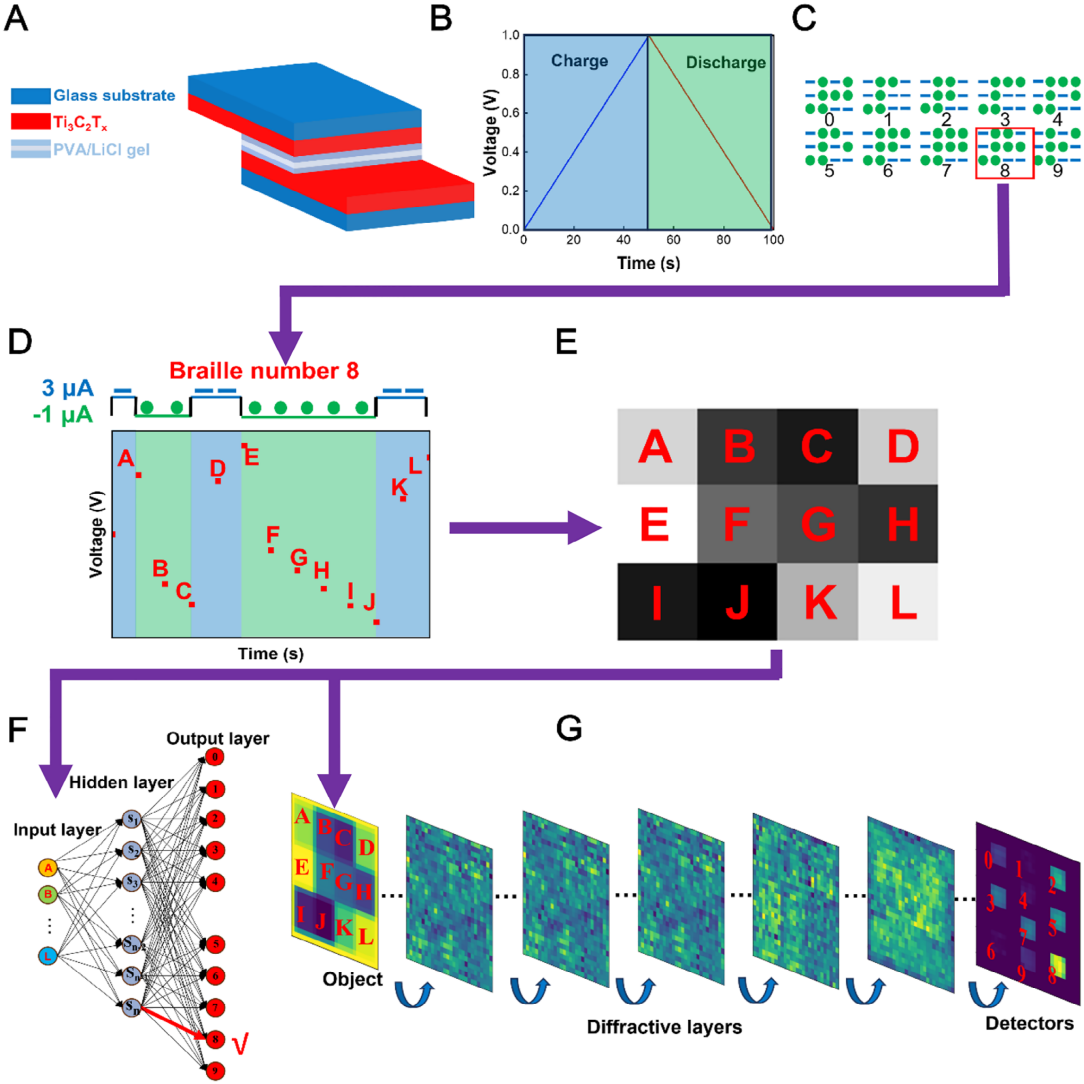
Mechanism of MXene Ti₃C₂T_x_ supercapacitor‐based neuromorphic computing. A) Structure of the supercapacitor. B) Voltage changes during charging/discharging procedures. C) Braille arrays representing numbers 0–9. D) A voltage curve of the supercapacitor stimulated by a current pulse train representing Braille number 8. E) A grayscale image of Braille number 8 converted by the voltage responses of the supercapacitor. F) An ANN classifying Braille number 8. G) A D^2^NN classifying Braille number 8.

The Scheme of MXene Ti₃C₂Tx supercapacitor‐based neuromorphic computing is shown in **Figure** **1**. Ti₃C₂Tx electrode films are separated by a polypropylene film, and PVA/LiCl gel electrolyte is added to form a sandwich structure for the double‐layer supercapacitor (Figure 1A). The voltage response increases as a forward current is applied to the supercapacitor. Then, it decreases under a reverse current (Figure 1B). The charging/discharging principle is used for Braille number recognition. Braille numbers 0–9 can be represented by a 3 × 4 array of hyphens and dots. For example, Braille number 8 has five hyphens and seven dots (Figure 1C). Next, we encode the 3 × 4 array to a current pulse train, in which the hyphen and the dot convert to a forward and a reverse current pulse, respectively (Figure 1D). Voltages collected at the end of each pulse are converted into grayscale values in direct ratio, and then a 12‐pixel image is generated as the representative of Braille number 8 (Figure 1E). Because voltage values differ under various current pulse trains, images of Braille numbers 0–9 can be classified using ANNs (Figure 1F) and D2NNs (Figure 1G).

### 2.2 Energy Storage Performances of MXene Ti₃C₂Tx Supercapacitors

**Figure 2:**
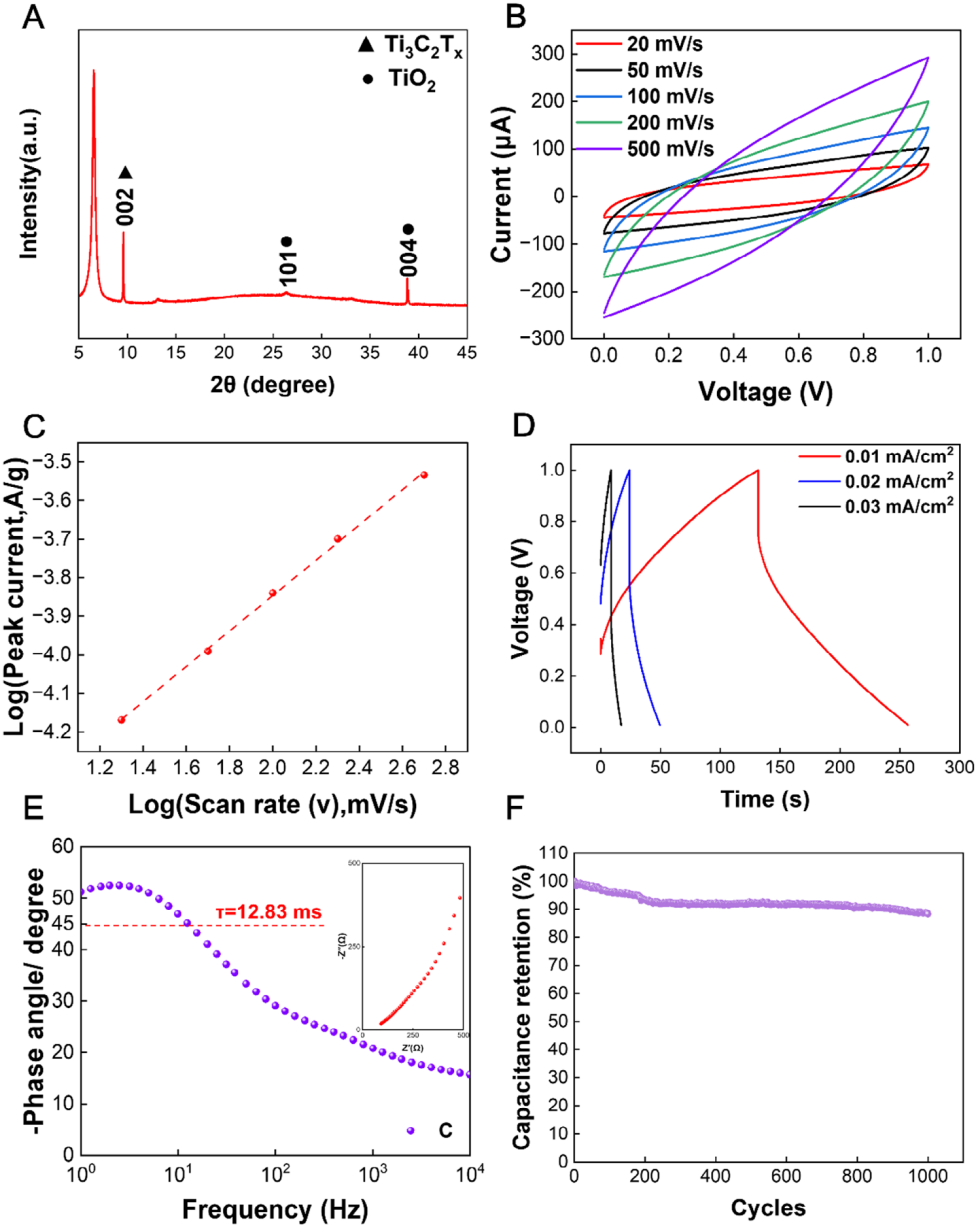
Energy storage performances of a MXene Ti₃C₂T_x_ supercapacitor. A) A XRD pattern of Ti₃C₂T_x_. B) CV curves of the supercapacitor at different scan rates. C) Logarithmic relationship between the current of the redox peaks and scan rate (20–500 mV s^−1^). D) GCD curves of the supercapacitor at various current densities. E) Phase angle plot and Nyquist plot (inset) for Ti₃C₂T_x_ supercapacitor. F) Electrochemical stability of the supercapacitor within 1000 charge/discharge cycles.

Ti₃C₂T_x_ is synthesized through a mild etching method using Ti_3_AlC_2_ powder as a precursor. A strong reflection peak at 2θ of 9.5° is observed in the X‐ray diffraction (XRD) pattern and matched with the single‐crystal data of Ti₃C₂T_x_, indicating that the as‐prepared material has a crystalline structure (**Figure** **2**A). In addition, peaks at 25° and 38° correspond to the (101) and (004) planes of TiO₂, which formation is due to the exposure of the sample to air during the reaction process.^[^ ^15^ ^]^ To observe surface morphology, a scanning electron microscopy (SEM) image of Ti₃C₂T_x_ was taken (Figure S1A, Supporting Information). This image shows nanosheet structures with a smooth surface. The layered structures contribute to a large specific surface area and enhance electrolyte infiltration, thus improving energy storage performances.^[^ ^16^ ^]^ Energy dispersive spectroscopy (EDS) of Ti₃C₂T_x_ displays the element distribution of carbon (C), oxygen (O), and titanium (Ti) (Figure S1B–D, Supporting Information).

The energy storage performances of the Ti₃C₂T_x_ supercapacitor are tested using an electrochemical workstation. Figure 2B shows the cyclic voltammetry (CV) curves at different scan rates from 20 to 500 mV s^−1^. The CV curves exhibit a prominent, nearly rectangular shape with notable humps at both ends of the voltage window, indicating a pseudocapacitive behavior. The specific capacitances at scan rates of 20 and 500 mV s^−1^ are 1696 and 288.5 µF cm^−^^2^, respectively. The CV curve with a voltage window from 0 to 1.2 V also exhibits a nearly rectangular shape (Figure S2, Supporting Information). Figure 2C illustrates the relationship between the peak current (*i*) and the scan rate (*v*). The data follows a power‐law equation: *i = a×v^b^*, where a and b are variables. A b value of 0.5 suggests that the charge storage process is primarily diffusion‐controlled, with significant pseudocapacitive behavior.^[^ ^17^ ^]^ Figure 2D displays voltage curves of galvanostatic charge‐discharge (GCD) tests at different current densities ranging from 0.01 to 0.03 mA cm^−2^. The specific capacitances increase with decreased current densities from 1.32 to 0.26 mF cm^−^^2^. To further investigate the electrochemical performance, electrochemical impedance spectroscopy (EIS) is conducted, as shown in Figure 2E. The calculated relaxation time, τ = 12.83 ms, is obtained when the phase angle is 45°. This indicates that the Ti₃C₂T_x_ supercapacitor exhibits strong ion diffusion and rapid charge transfer properties in the low‐frequency region. The Nyquist plot inset also reveals a sloping increase in impedance in the low‐frequency region. This suggests that the electrochemical reaction is primarily controlled by ion diffusion, aligning with the findings in Figure 2C. Next, a 0.02 mA cm^−2^ current density is selected for a 1000‐cycle charge/discharge test to observe the electrochemical stability. Due to the prominent pseudocapacitive effect, the irreversible oxidation of Ti₃C₂T_x_ electrode material leads to capacity degradation.^[^ ^18^ ^]^ As shown in Figure 2F, the supercapacitor remains at 88% of the initial capacitance after 1000 cycles. When the voltage window is from 0.08 to 0.12 V, the capacity retention after 5000 charge‐discharge cycles is 98.16%, as shown in Figure S3 (Supporting Information).

### 2.3 Synaptic Plasticity of Supercapacitors

**Figure 3:**
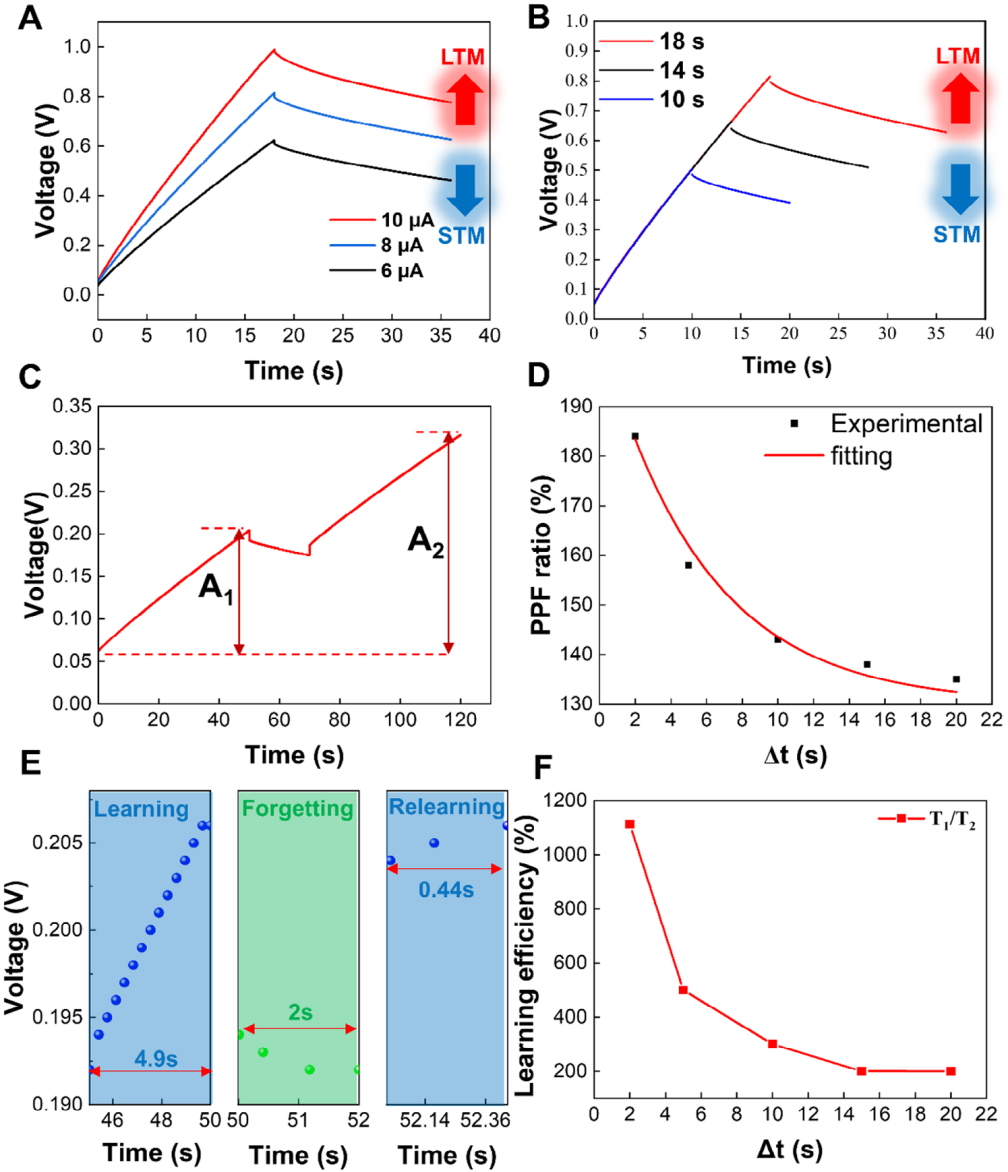
Synaptic plasticity of a MXene Ti₃C₂T_x_ supercapacitor. A) STM‐LTM transition under different charging currents and a discharging current of ‐1 µA. B) STM‐LTM transition under different charging durations and a discharging current of ‐1 µA. C) PPF under a 20 s discharging interval between the two charging current stimuli. The charging and discharging currents are 3 and ‐1 µA. D) PPF index changes under different intervals between the two charging current stimuli. E) Learning experience under charging pulses of 3 µA and a discharging pulse of ‐1 µA. F) Learning efficiency changes under different durations of the forgetting process.

Voltage responses of supercapacitors under current stimulation can mimic synapse behaviors. The memory of biology synapses can be divided into STM and LTM.^[^ ^19^ ^]^ STM is stored in the hippocampus and transferred to the cerebral cortex. Under stimulation with high intensities and long durations, STM can turn into LTM. To investigate the relationship between voltage responses and current stimulation strength, a charging current pulse and a discharging current pulse are applied (**Figure** **3**A). As the charging currents increase from 6 to 10 µA, the voltage responses become higher, indicating a transition from STM to long‐term memory through intensive stimulation. Enhancing the current stimulation duration can also realize the STM‐LTM transition. As shown in Figure S4 (Supporting Information). To characterize the dynamic voltage decay behavior after the current removal, the decay process is fitted using the Kohlrausch stretched exponential function.^[^ ^20^ ^]^

(1) $$R_{t}=\Delta R\;\exp\;{\left(-\frac{t}{\tau }\right)}^{\beta }+\cdot R_{0}$$

yielding a relaxation time of τ = 144 s and a stretching exponent β = 0.11. The τ value indicates that the material possesses good nonvolatility, making it suitable for tunable memory response characteristics. The voltage responses enhance as the charging durations increase from 10 to 18 s (Figure 3B). The results imply that the synaptic plasticity of supercapacitors can be tuned by adjusting charging currents and durations.

PPF, another typical behavior of biological synapses in which a second presynaptic stimulation induces a higher postsynaptic response than the first stimulation,^[^ ^21^ ^]^ is successfully mimicked by the voltage responses of the supercapacitors. Figure 3C shows the PPF behavior of Ti₃C₂T_x_ supercapacitors under a 20 s discharging interval between the two charging current stimuli. The voltage response after the second stimulation (A_2_) is more significant than after the first pulse (A_1_). The PPF behavior under the interval ranging from 2 to 15 s is shown in Figure S5 (Supporting Information). Figure 3D presents the relationship between PPF indexes and stimulation intervals. The PPF index defined by A_2_/A_1_ is inversely proportional to the interval between the stimuli. As the interval increases from 2 to 20 s, the PPF index decreases from 184% to 135%. The decay in the PPF index can be described using a double‐exponential function:

(2) $$\mathrm{PPF}\;\mathrm{index}=1+c_{1}\;\exp\left(-\frac{\Delta t}{\tau_{1}}\right)+C_{2}\;\left(-\frac{\Delta t}{\tau_{2}}\right)$$

where *C_1_* and *C_2_* are the initial facilitation values of the two stimuli, and are the characteristic relaxation times of the respective phases. is fitted to be 3.2 s of 283.2 s is about orders of magnitude larger than, which is similar to the phenomenon in biological synapses.^[^ ^19^ ^]^

Based on the synaptic plasticity, the supercapacitor can mimic the human's learning experience – less time is required to reach the same level of cognition when the same thing is learned again.^[^ ^22^ ^]^ As shown in Figure 3E, the first stimulation of the charging current enhances the voltage from 0.192 to 0.206 V (learning process). Then, a discharging process decreases the voltage to 0.192 V with a significant IR drop (forgetting process). The details of the IR drop are shown in Figure S6 (Supporting Information). The measurement indicates that (relearning process) the first exposure requires 4.9 s (*T_1_*) to complete the voltage decrease, whereas the second stimulation leading to the exact change takes only 0.44 s (*T_2_*). The values of *T_1_* and *T_2_* under various durations of the forgetting process are shown in Figure S7 (Supporting Information). Learning efficiency, defined by *T_1_*/*T_2_ × 100%*, is inversely proportional to the forgetting duration (Figure 3F). Because of the significant voltage change at the beginning of the relearning process, all the learning efficiencies are above 100%. The highest learning efficiency reaches 1113%.

### 2.4 Supercapacitor‐Based Neuromorphic Computing Using ANNs and D2NNs

**Figure 4:**
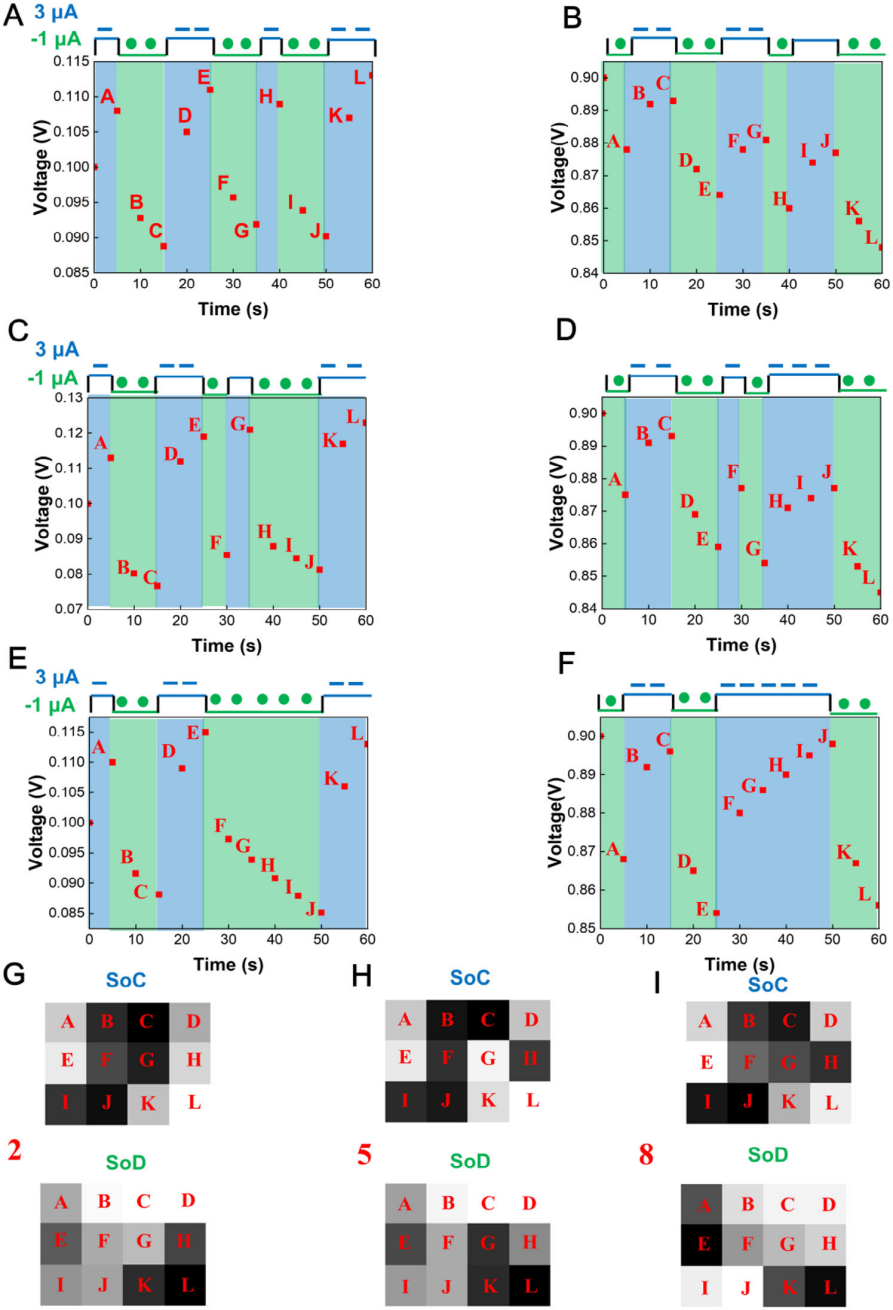
Generation of images for Braille numbers 0–9 as inputs of neuromorphic computing. Voltage curves for Braille number 2 in SoC (A) and SoD (B), Braille number 5 in SoC (C) and SoD (D), and Braille number 8 in SoC (E) and SoD (F). Grayscale images for Braille numbers 2 (G), 5 (H), and 8 (I).

The synaptic plasticity of supercapacitors indicates that tunable voltage responses can be achieved through charging/discharging processes and has excellent potential for neuromorphic computing. Herein, we demonstrate supercapacitor‐based neuromorphic computing for recognizing Braille numbers 0–9. Each Braille number is a 3 × 4 array of hyphens and dots (Figure 1C). The array is encoded to a charging/discharging pulse train. The pulse train is applied to supercapacitors through two pathways – SoC and SoD. For SoC, the hyphen and the dot represent a charging current stimulation of 3 µA and a discharging current stimulation of ‐1 µA, respectively. The initial voltage of the supercapacitor is 0.1 V. For SoD, the hyphen, and the dot represent a discharging current stimulation of −1 µA and a charging current stimulation of 3 µA, respectively. The initial voltage of the supercapacitor is 0.9 V. The voltage values at the end of each pulse are measured using an electrochemical workstation, as shown in **Figure** **4**A–F and Figures S8 and S9 (Supporting Information). An excellent cyclic stability is demonstrated within the voltage windows (Figure S3, Supporting Information). Next, the voltage values are converted into a 12‐pixel grayscale image. As shown in Figure 4G–I, grayscale images corresponding to Braille numbers 2, 5, and 8 in SoC and SoD are all different. The images for other Braille numbers are shown in Figures S10 and S11 (Supporting Information). These images serve as inputs for ANN and D^2^NN models. Through the voltage measurement involving a Ti₃C₂T_x_ supercapacitor, we notice that the standard deviation of the digit “2” at point A in Figure 4A is 0.001. To better adapt to real‐world scenarios, different levels of Gaussian noise (0.1, 0.5, and 1) are added to the ANN and D^2^NN models.

**Figure 5:**
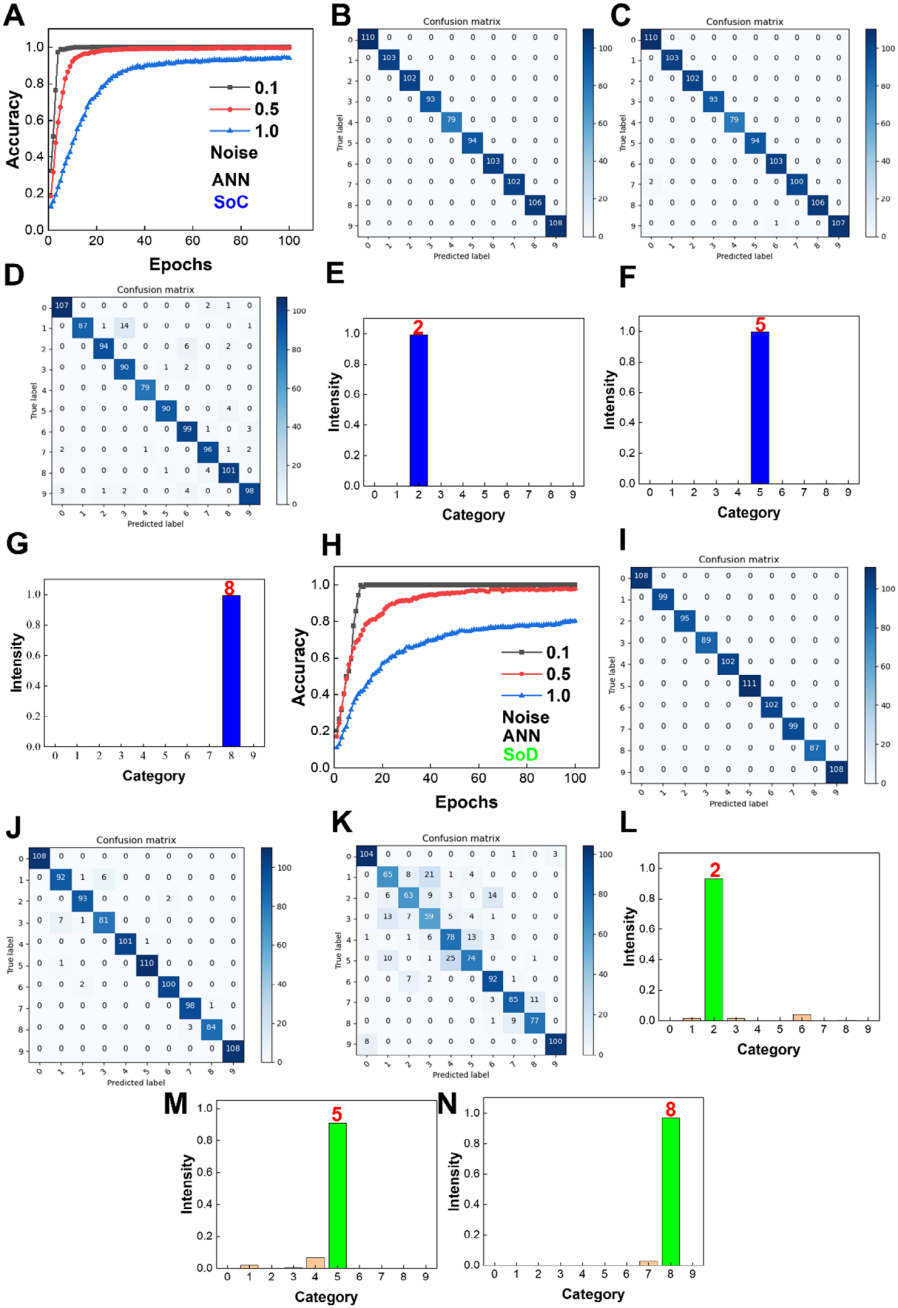
Supercapacitor‐based neuromorphic computing using an ANN for recognition of Braille numbers. A) Recognition accuracies in SoC with different standard deviations of Gaussian noises. Confusion matrices under 0.1 (B), 0.5 (C), and 1.0 (D) deviations of Gaussian noises in SoC. Output intensity distributions of Braille numbers 2 (E), 5 (F), and 8 (G) in SoC. The noise deviation is 0.5. H) Recognition accuracies in SoD with different standard deviations of Gaussian noises. Confusion matrices under 0.1 (I), 0.5 (J), and 1.0 (K) deviations of Gaussian noises in SoD. Output intensity distributions of Braille numbers 2 (L), 5 (M), and 8 (N) in SoD. The noise deviation is 0.5.

High recognition accuracies are realized through ANN processing of the images for Braille numbers. For the SoC, Gaussian noises with three levels of standard deviations are added. With a deviation of 0.1, the ANN achieves 100% Braille classification accuracy at the 18th epoch. With a deviation of 0.5, the accuracy reaches 99.7% at the 100th epoch. With a deviation of 1.0, the accuracy is 94.1% after 100 epochs (**Figure** **5**A). Figure 5B–D shows confusion matrices at 100 epochs under different noise levels. Under low‐deviation noise, all braille numbers are accurately identified. Under medium and high‐deviation noises, most numbers are still correctly classified, although some misclassifications occur. Figure 5E shows the output intensity distribution of Braille number 2. Due to the maximum intensity distribution of number 2, the ANN successfully recognizes the image as number 2. The output intensity distributions of other Braille numbers are illuminated in Figure 5F,G, and Figure S12 (Supporting Information). For SoD, as shown in Figure 5H, with a noise deviation of 0.1, the ANN achieves 100% accuracy at the 13th epoch. With deviations of 0.5 and 1.0, the accuracies reach 97.7% and 80.1% after 100 epochs, respectively. Figure 5I–K shows confusion matrices at 100 epochs under different noise levels. Figure 5L–N and Figure S13 (Supporting Information) demonstrate output intensity distributions. All the results verify that ANN processing of supercapacitor‐generated signals can recognize Braille numbers with high accuracies. For comparison, a graphene electrical double‐layer capacitor is fabricated through laser scribing.^[^ ^23^ ^]^ GCD tests show linear curves with marginal IR drops (Figure S14A, Supporting Information). Under an interval of 2 s, the learning efficiency of the graphene‐based supercapacitor is 150%, much lower than that of the Ti₃C₂T_x_ supercapacitor (Figure S14B, Supporting Information). With a noise deviation of 0.1, the recognition accuracy for Braille is 99.8% after 50 epochs, which is also lower than that of the Ti₃C₂T_x_ supercapacitor (Figure S14C, Supporting Information). The marginal IR drops lead to similar voltage changes during different discharging pulses (“dot” pulses). The lack of specific voltage responses induces relatively low recognition accuracy.

The voltage curves of supercapacitors for Braille numbers are time‐dependent and thus advantageous to achieving high recognition accuracies through recurrent neural networks (RNNs).^[^ ^24^ ^]^ The accuracies for recognition of numbers 0–9 in SoC and SoD are shown in Figure S15 (Supporting Information). The validation accuracies reached 100% at the 8th epoch in both SoC and SoD. Compared to the ANN, the RNN reached 100% accuracy earlier in both SoC and SoD. However, the accuracy in SoD fluctuates due to the significant voltage drop.

**Figure 6:**
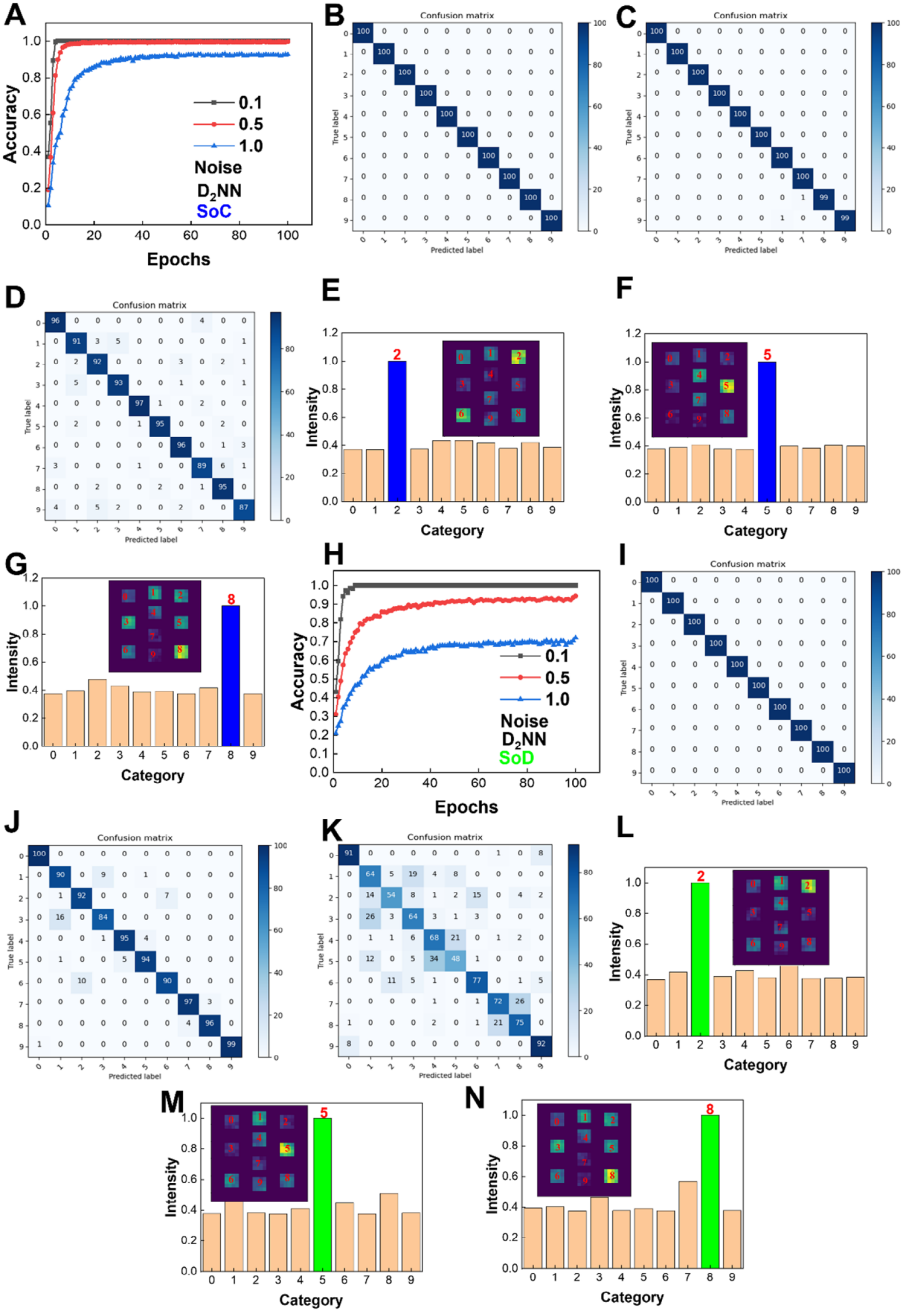
Supercapacitor‐based neuromorphic computing using a D^2^NN for recognition of Braille numbers. A) Recognition accuracies in SoC with different standard deviations of Gaussian noises. Confusion matrices under 0.1 (B), 0.5 (C), and 1.0 (D) deviations of Gaussian noises in SoC. Output intensity distributions of Braille numbers 2 (E), 5 (F), and 8 (G) in SoC. The noise deviation is 0.5. H) Recognition accuracies in SoD with different standard deviations of Gaussian noises. Confusion matrices under 0.1 (I), 0.5 (J), and 1.0 (K) deviations of Gaussian noises in SoD. Output intensity distributions of Braille numbers 2 (L), 5 (M), and 8 (N) in SoD. The noise deviation is 0.5.

D^2^NNs are all‐optical neural networks combining the principle of optical diffraction with deep learning and are notable for excellent performance in image recognition.^[^ ^25^ ^]^ **Figure** **6**A shows the Braille recognition accuracy of a D^2^NN model under different Gaussian noise deviations in SoC. At a noise deviation of 0.1, the D^2^NN achieves 100% accuracy at the 5th epoch, better than an ANN. Under deviations of 0.5 and 1.0, the accuracies reach 99.8% and 92.8% after 100 epochs, respectively. Figure 6B–D shows the confusion matrices in SoC after 100 epochs under various noise deviations. Output intensity distributions of Braille numbers are shown in Figure 6E–G and Figure S16 (Supporting Information). These results further verify the high‐accuracy recognition. For SoD, at a low noise deviation of 0.1, the D^2^NN achieves 100% accuracy at the 9th epoch. At medium and high deviations of 0.5 and 1.0, the accuracies after 100 epochs are 94.5% and 72.1%, respectively (Figure 6H). Compared to the SoC, the SoD shows slightly more misclassified digits due to the combined effects of noise and voltage drop. The recognition results can also be verified through confusion matrices and output intensity distributions (Figure 6I–N and Figure S17, Supporting Information). Since D^2^NNs are designed to recognize images with at least several hundred pixels and the image representing a Braille number contains only 12 pixels, D^2^NN reaches 100% accuracy faster under a low noise deviation than an ANN.^[^ ^26^ ^]^

To further explore the minimum response time and energy of the supercapacitor‐based neuromorphic computing, a short current pulse (0.23 µA, 0.13 s duration) is applied. A significant voltage response of 26.29 mV can be observed. During the pulse, 0.79 nJ is stored in the supercapacitor, using the formula E = V × I × t (Figure S18A, Supporting Information). Feeding time‐dependent voltage responses and baseline data without pulses into an RNN model achieves 100% classification accuracy for the voltage response recognition after 18 training epochs (Figure S18B, Supporting Information). The energy issue is crucial for neuromorphic computing. Because energy is stored during the state of charge in supercapacitors and released during the state of discharge, supercapacitors have great potential to cut the energy consumption of signal conversion during neuromorphic computing.

## 3 Conclusion

This study presents a supercapacitor‐based pathway of neuromorphic computing in which the electrical responses of supercapacitors serve as the computing input. Energy is stored during the response enhancement and released once the response declines, thus avoiding high energy consumption when generating the input signals. Ti₃C₂T_x_ supercapacitor electrodes are fabricated using Ti_3_AlC_2_ powder as a precursor. The supercapacitor exhibits a specific capacitance of 1.32 mF cm^−2^ under a current density of 0.01 mA cm^−2^. Voltage responses of the supercapacitors are tunable under different parameters of current stimulation, and thus, synaptic behavior can be mimicked. The current values and durations determine the plasticity of voltage responses during charging/discharging processes, demonstrating synaptic behaviors, including STM‐LTM transition, PPF, and learning experience and potential to recognize Braille numbers through neuromorphic computing. The Braille numbers are encoded to charging/discharging current pulse trains, and then the pulse trains are applied to supercapacitors to collect voltage responses. The neuromorphic computing inputs are generated through a conversion from the responses to greyscale images. An ANN classifies Braille numbers 0–9 with 100% accuracy under a low noise deviation. A D^2^NN reaches 100% accuracy even faster than the ANN. The results lay a solid foundation for designing energy‐efficient, high‐performance neuromorphic computing hardware.

## 4 Experimental Section

### Materials

Titanium aluminum carbide (Ti_3_AlC_2_, mass fraction ≥90%) was purchased from Nanjing Xianfeng Nano Material Technology. Lithium chloride (LiCl, analytical grade), acetone (C_3_H_6_O, analytical grade), anhydrous ethanol (C_2_H_6_O, analytical grade), lithium fluoride (LiF, analytical grade), and hydrochloric acid (HCl, mass fraction 36.0%‐38.0%) were purchased from Sinopharm Chemical Reagent.

Preparation and Characterization of Ti3C2Tx: Ti3C2Tx was synthesized through a mild etching method using Ti_3_AlC_2_ powder as a precursor. First, 1.62 g of LiF was added to 40 mL of diluted 9 mol L^−1^ HCl and stirred. The solution was heated to 35 °C to ensure thorough mixing. Subsequently, 1 g of Ti_3_AlC_2_ was slowly added to the solution. After 24 h reaction, the sediment was centrifuged and repeatedly washed with deionized water until the pH was ≥6. Finally, 35 mL of deionized water was mixed to prepare 10 g L^−1^ Ti_3_C_2_T_x_ suspension. Next, 1 mL of Ti_3_C_2_T_x_ suspension was drop‐cast onto a glass substrate using a drop‐casting method. The substrate was left in a fume hood at room temperature for 24 h to form a film. The Ti_3_C_2_T_x_ was observed using a Zeiss Sigma 300 SEM and was measured using a Bruker D8 Advance XRD.

### Assembly and Characterization of Ti_3_C_2_T_x_ Supercapacitors

1 g of PVA was added to 10 mL of deionized water, and then 2 g of LiCl was added to prepare a 4.72 mol L^−1^ PVA/LiCl gel electrolyte. The electrode film was divided into two parts, leaving a 10 mm × 10 mm square at one end. A single‐layer polypropylene separator (Celgard2500, 25µm) was cut into slightly larger squares. The separator was soaked with the PVA/LiCl gel electrolyte and sandwiched between the electrode films. The assembled supercapacitor was then electrochemically tested using a Garmy Interface 1010E electrochemical workstation. EIS was performed at an AC voltage amplitude of 5 mV in the frequency range of 0.01–100 kHz. EIS data was fitted to an electrical equivalent circuit model using the software ZView.

### Neuromorphic Computing

Braille numbers 0–9 were encoded to current pulse trains. An initial voltage of 0.1V was SoC and 0.9V was defined as SoD. In SoC, a charging current pulse of 3 µA was used to simulate hyphen symbols in the Braille array, and a ‐1 µA discharging pulse was used to simulate dot symbols. In SoD, a ‐3 µA discharging pulse and a 1 µA charging pulse were used to simulate hyphen and dot symbols. Each pulse was applied for 5 s. The voltage values were converted into a 3 × 4 grayscale image with grayscale values ranging from 0 to 255. For each pulse train, 500 images were generated through the experiment results of the voltage values. The dataset contains 5000 images in total, with 80% for training and 20% for testing. For ANN‐based neuromorphic computing, a multilayer perceptron (MLP) model was designed to recognize Braille numbers 0–9. The model consists of four layers: the input layer contains 784 neurons, the two hidden layers have 128 and 64 neurons respectively, and the output layer contains 10 neurons. Gaussian noise with standard deviations of 0.1, 0.5, and 1.0 were added. For RNN‐based neuromorphic computing, two long short‐term memory (LSTM) networks and dropout layers were used in the model's recurrent layers. The first and second LSTM layers contained 15 and 20 memory units, respectively. The model was trained using the Adam optimizer and mean square error loss function. The batch size was set to 15. For D^2^NN‐based neuromorphic computing, a distance of 10 millimeters between the diffractive plates and a light wavelength of 532 nanometers were applied. The D^2^NN was constructed with five 3D‐printed diffractive layers (The input layer and each diffraction layer contain 784 neurons, while the output layer consists of 10 neurons for classification.). The modulation characteristics of each layer were optimized using ReLU functions and normalization operations to ensure signal fidelity and computational efficiency. The training parameters included 100 epochs and a batch size of 10, with a learning rate set at 0.0001. The Adam optimizer was employed to minimize the cross‐entropy loss function.

## Conflict of Interest

The authors declare no conflict of interest.

## Author Contributions

X.C. conceived the idea, designed the experiments, and supervised the research. L.W. fabricated supercapacitors, performed characterizations and simulations, and analyzed neuromorphic computing data. X.L., G.Z., and F.Q. assisted in the supercapacitor fabrication and data processing. All authors contributed to the discussion and the manuscript writing.

## Supporting information



Supporting Information

## Data Availability

The data supporting this study's findings are available from the corresponding author upon request.
