# Supplementary material for: Neuromorphic Computing Using Synaptic Plasticity of Supercapacitors
Source: Adv Sci (Weinh). 2025 Mar 24;12(19):2500521. doi: 10.1002/advs.202500521 (PMC12097122; doi:10.1002/advs.202500521)
Supplement: Supplementary file 1 — Supporting Information [file ADVS-12-2500521-s001.docx]

**Supporting Information**

**Neuromorphic Computing using Synaptic Plasticity of Supercapacitors**

*Ling Wang, Xing Liu, Guangcai Zhang, Fuxun Qi, and Xi Chen**

L. Wang, X. Liu, G. Zhang, X. Chen

School of Artificial Intelligence Science and Technology, University of Shanghai for Science and Technology, Shanghai 200093, China

L. Wang, X. Liu, G. Zhang, X. Chen

Institute of Photonic Chips, University of Shanghai for Science and Technology, Shanghai 200093, China

L. Wang, F. Qi

School of Materials and Chemistry, University of Shanghai for Science and Technology, Shanghai, China, 200093

E-mail: xichen@usst.edu.cn


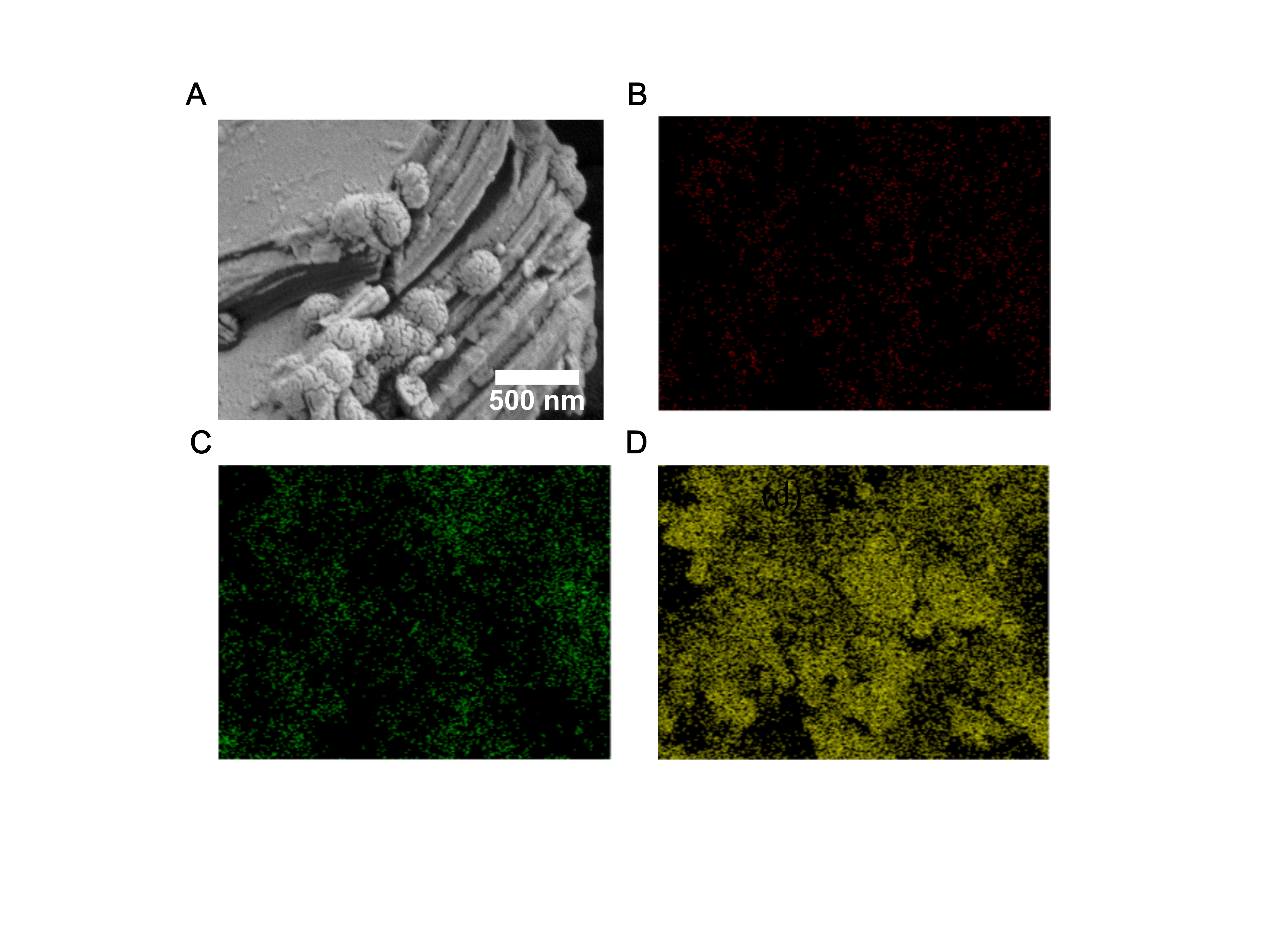


**Figure S1**. A) A SEM image of Ti_3_C_2_T_x_. Elemental distribution of Ti (B), C (C), and O (D) in Ti_3_C_2_T_x_.


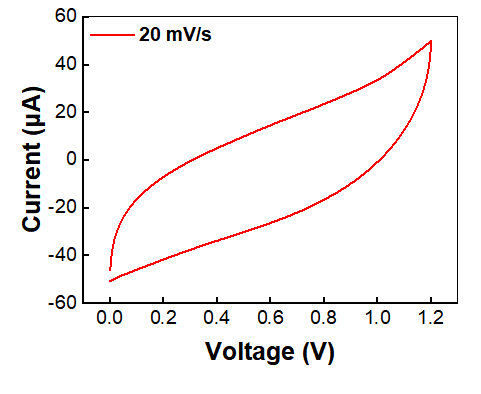


**Figure S2**. A CV curve of a MXene Ti₃C₂T_x_ supercapacitor with a voltage window from 0 V to 1.2 V.


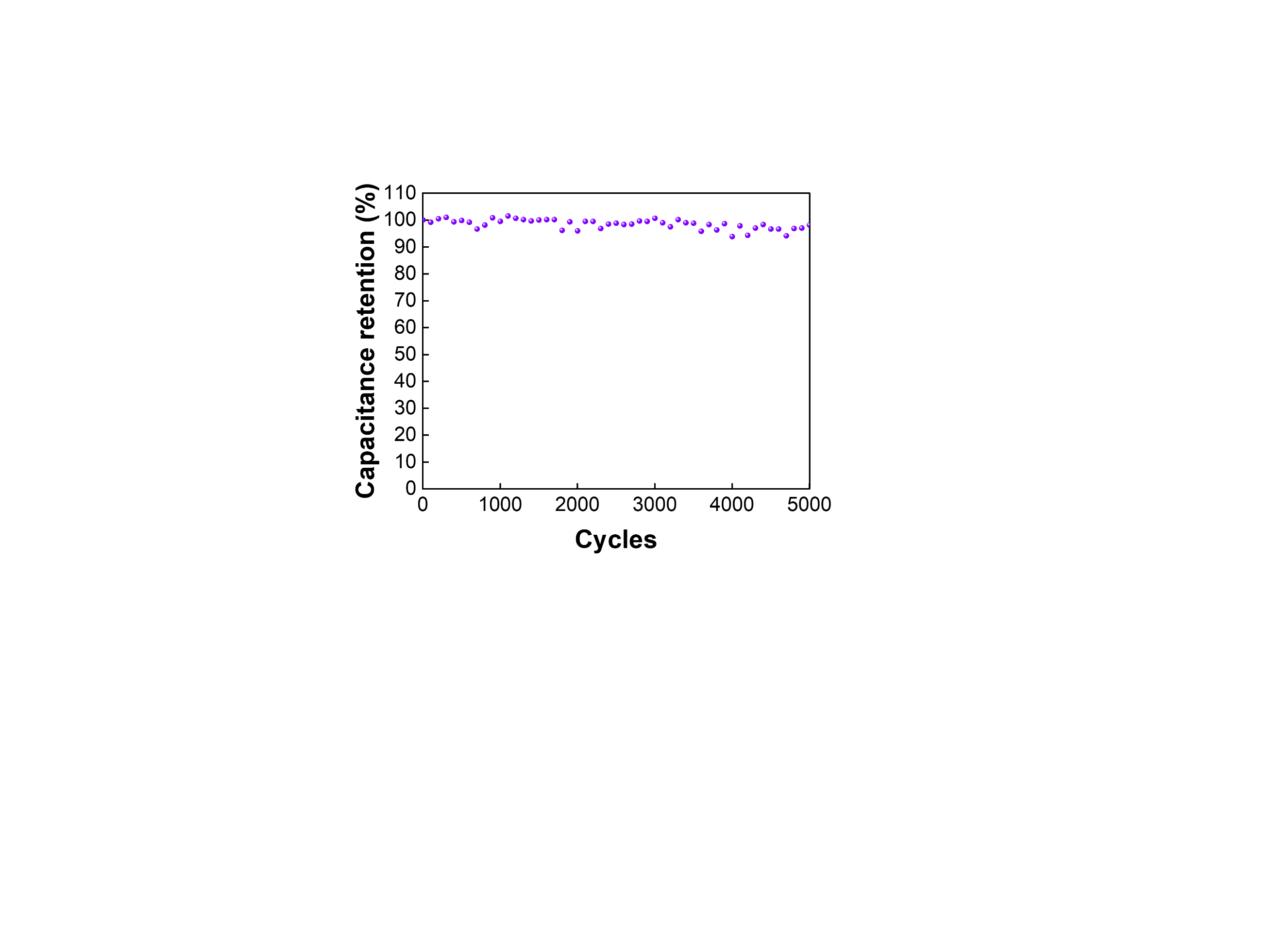


**Figure S3**. **Cycling stability of a Ti₃C₂T_x_ supercapacitor within a voltage window from 0.08 to 0.12 V.**


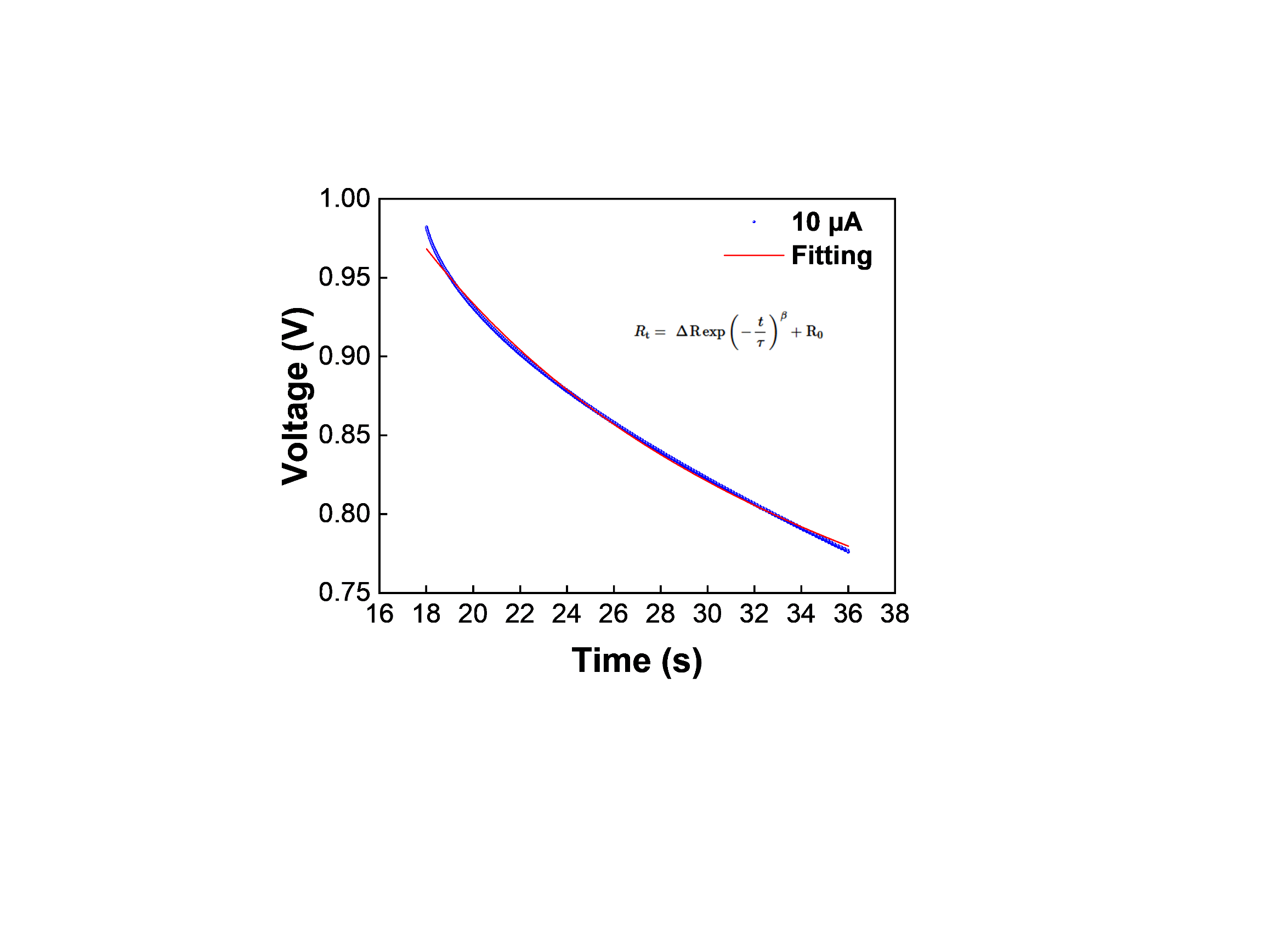


**Figure S4**. Voltage decay fitting curve at 10 μA current.

**
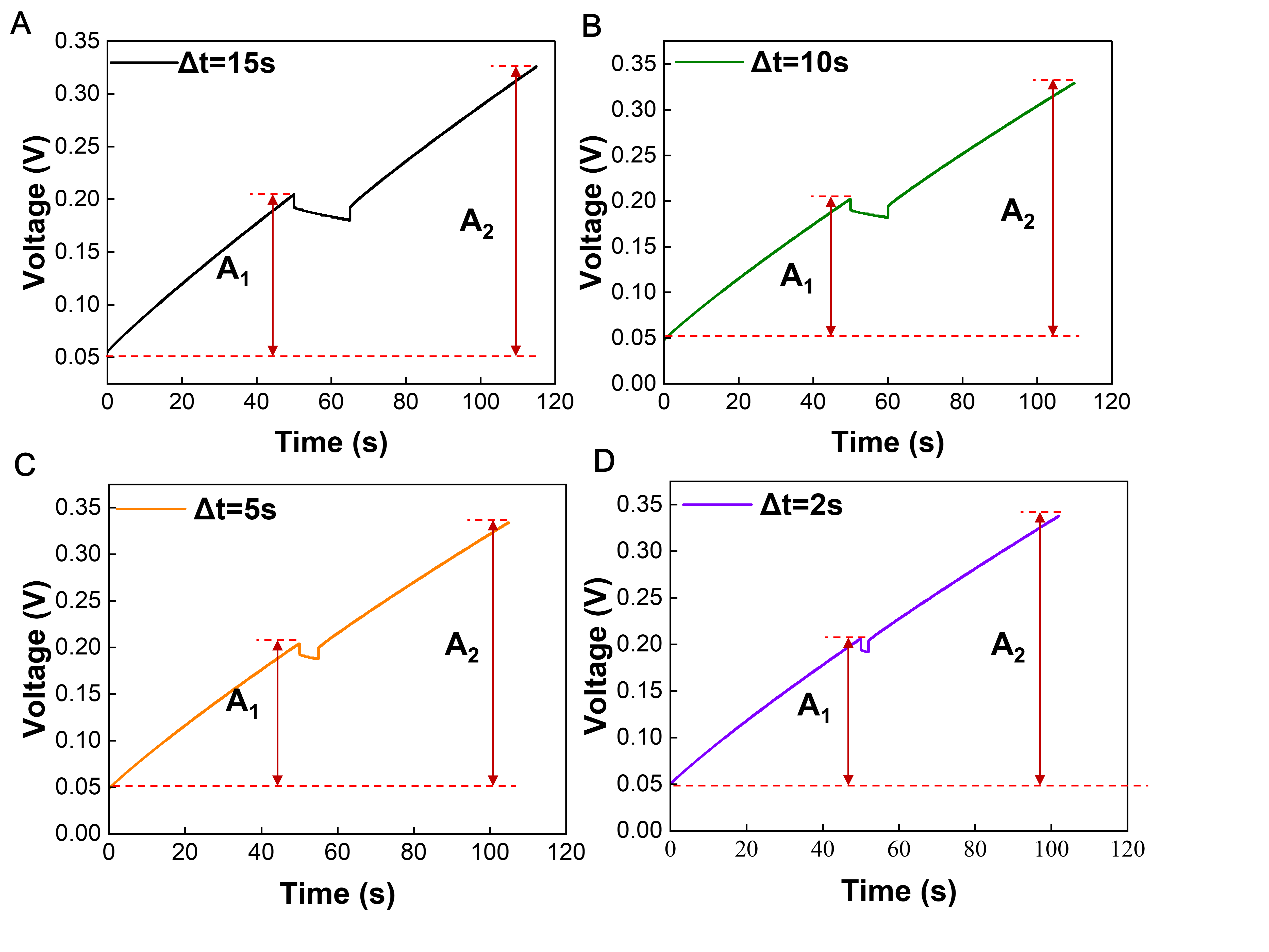
**

**Figure S5**. PPF under a discharging interval of 15 s (A), 10 s (B), 5 s (C), and 2 s (D) between the two charging current stimuli. The charging and discharging currents are 3 μA and -1 μA.


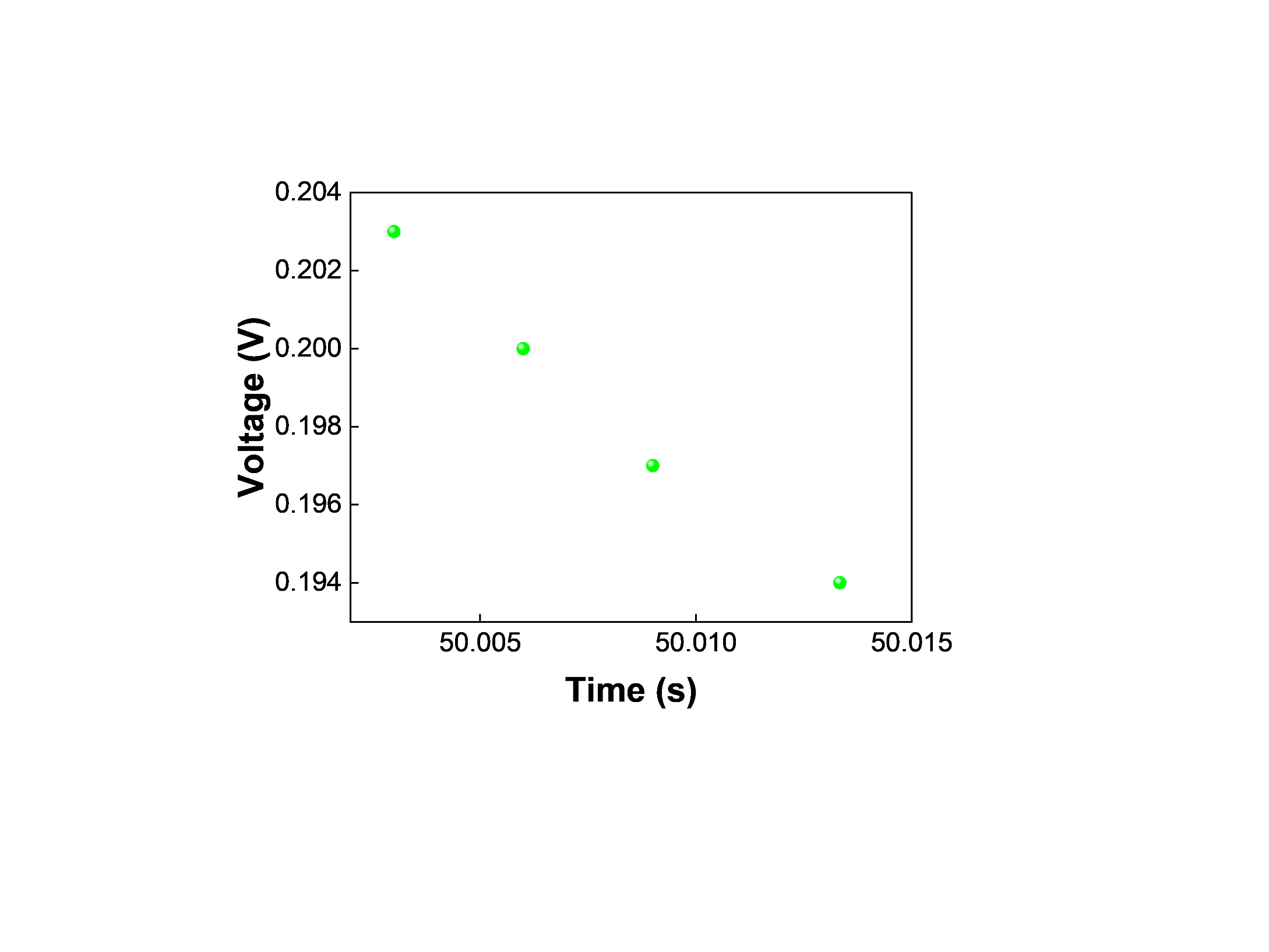


**Figure S6**. The details of IR drop in the forgetting process under a discharging pulse of -1 μA.


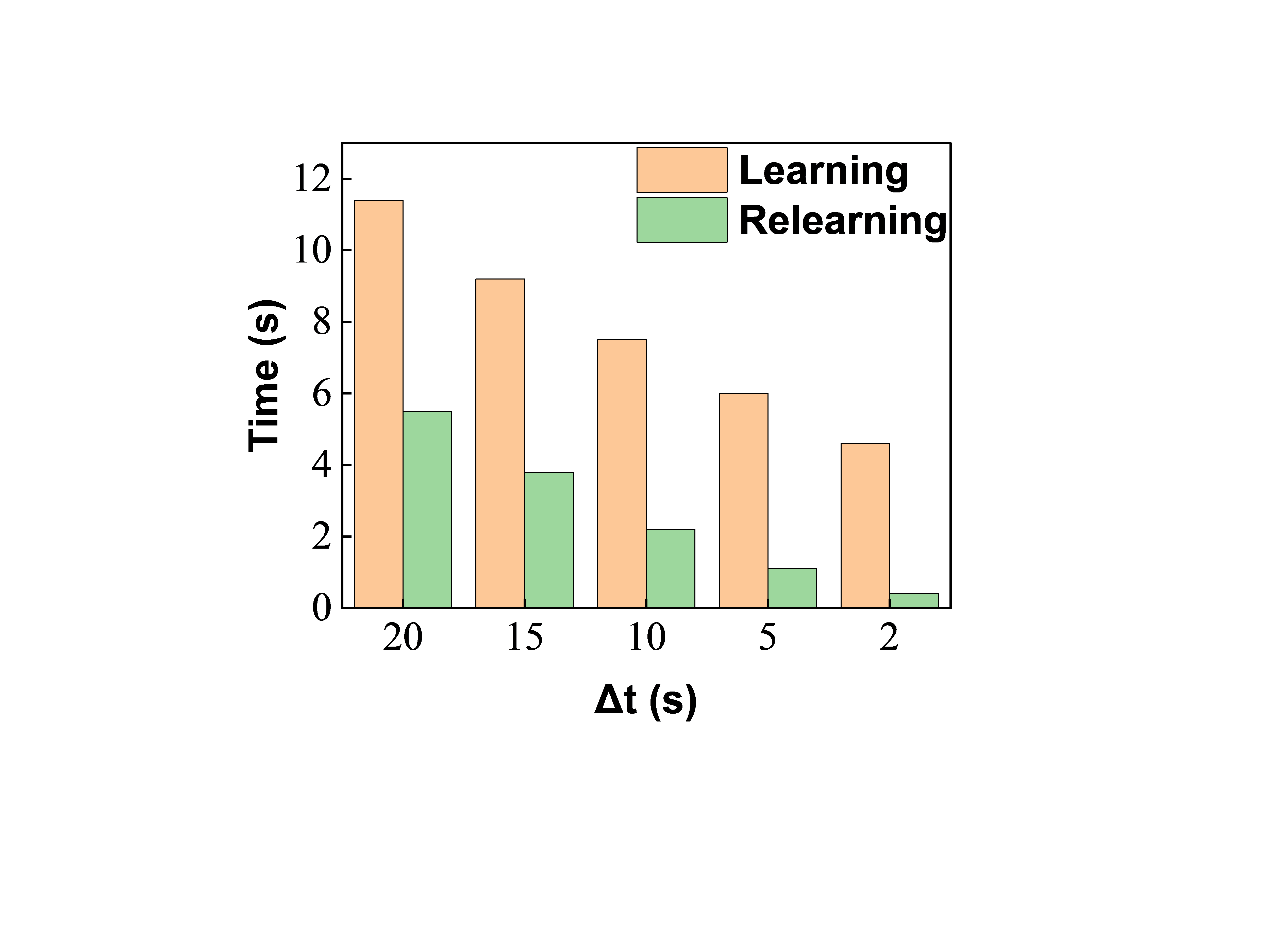


**Figure S7**. Values of learning durations and relearning durations under various durations of the forgetting process.

**
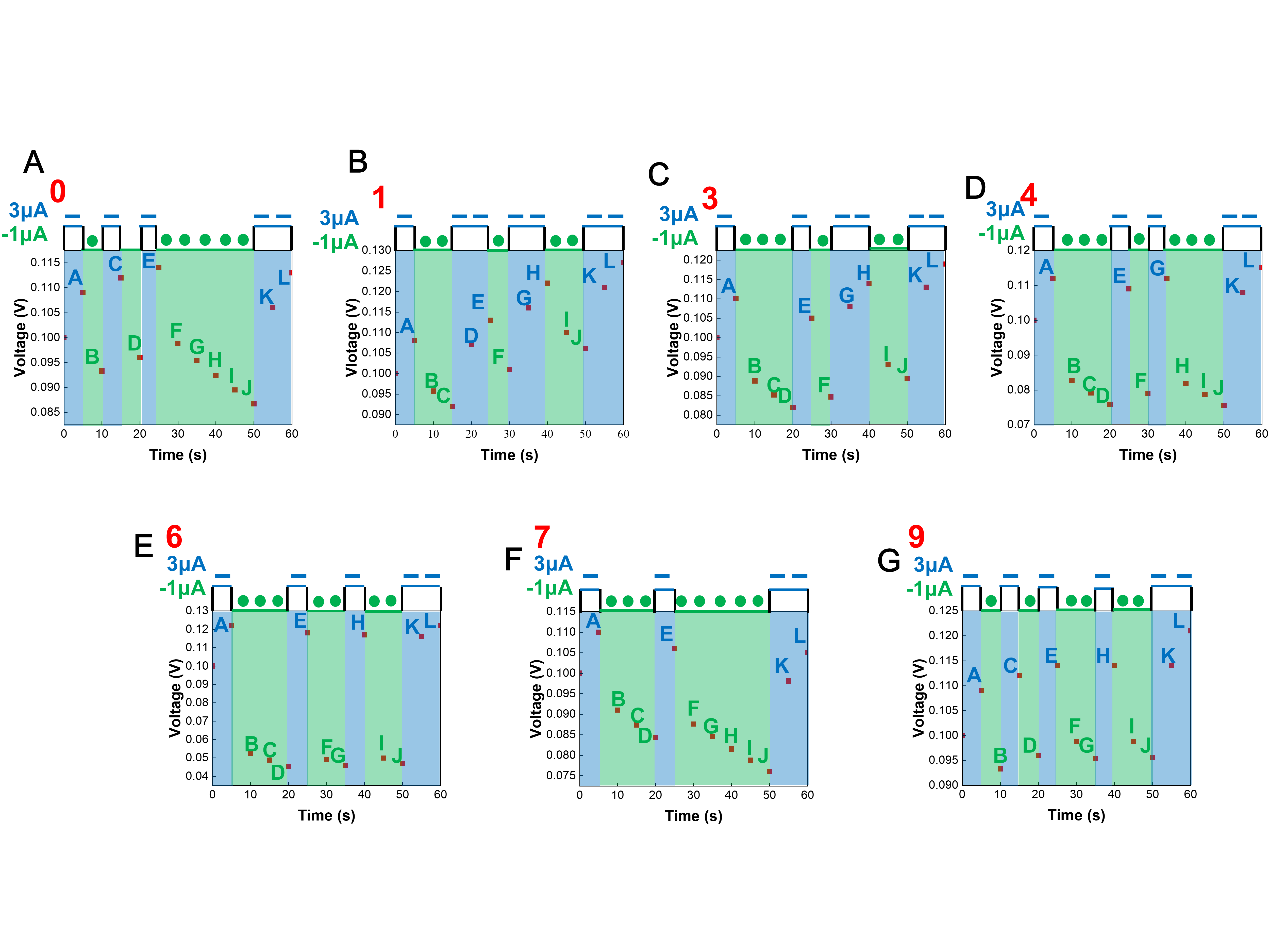
**

**Figure S8**. Voltage curves in SoC for Braille numbers 0, 1, 3, 4, 6, 7, and 9.

**
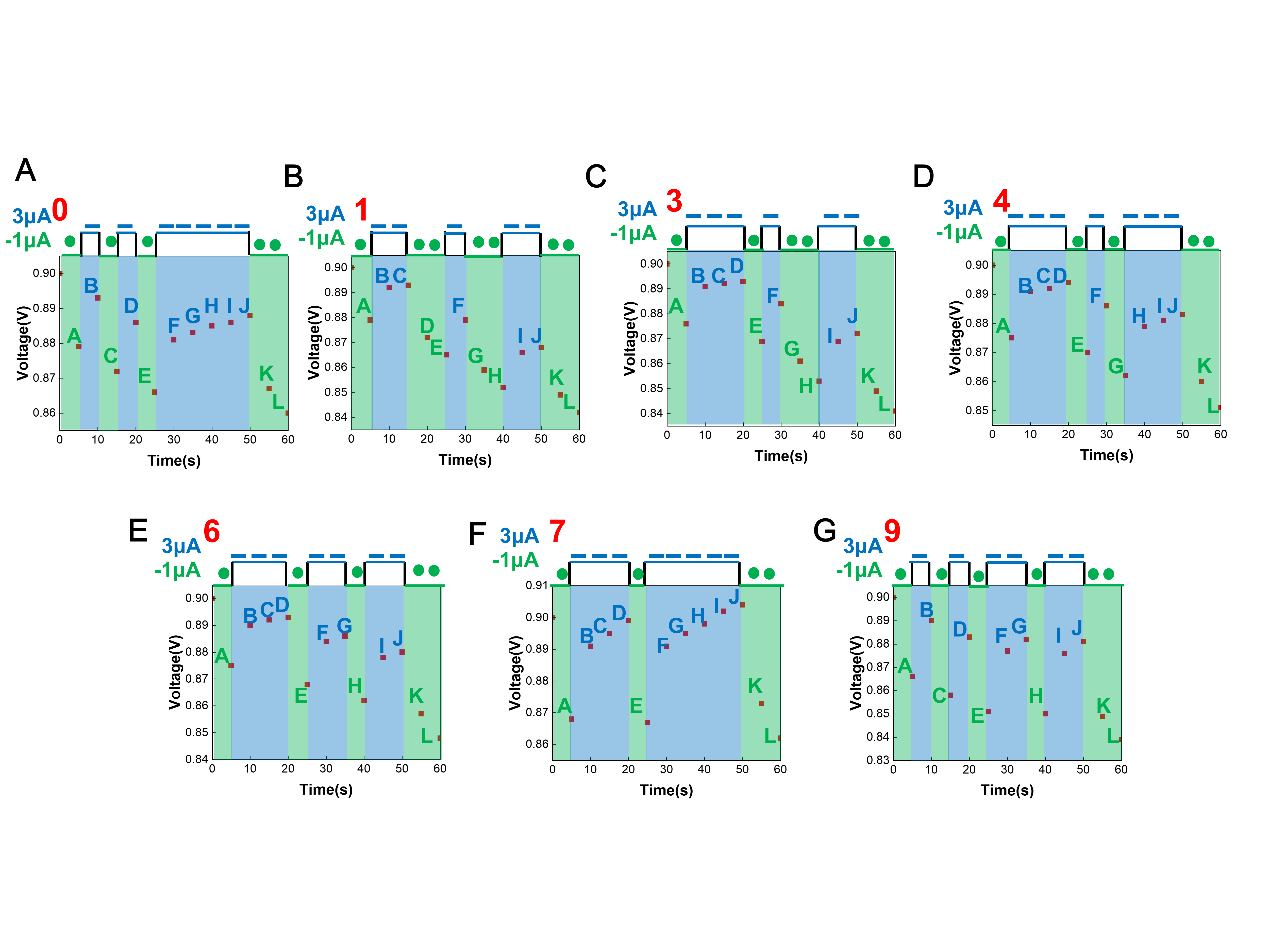
**

**Figure S9**. Voltage curves in SoD for Braille numbers 0, 1, 3, 4, 6, 7, and 9.

**
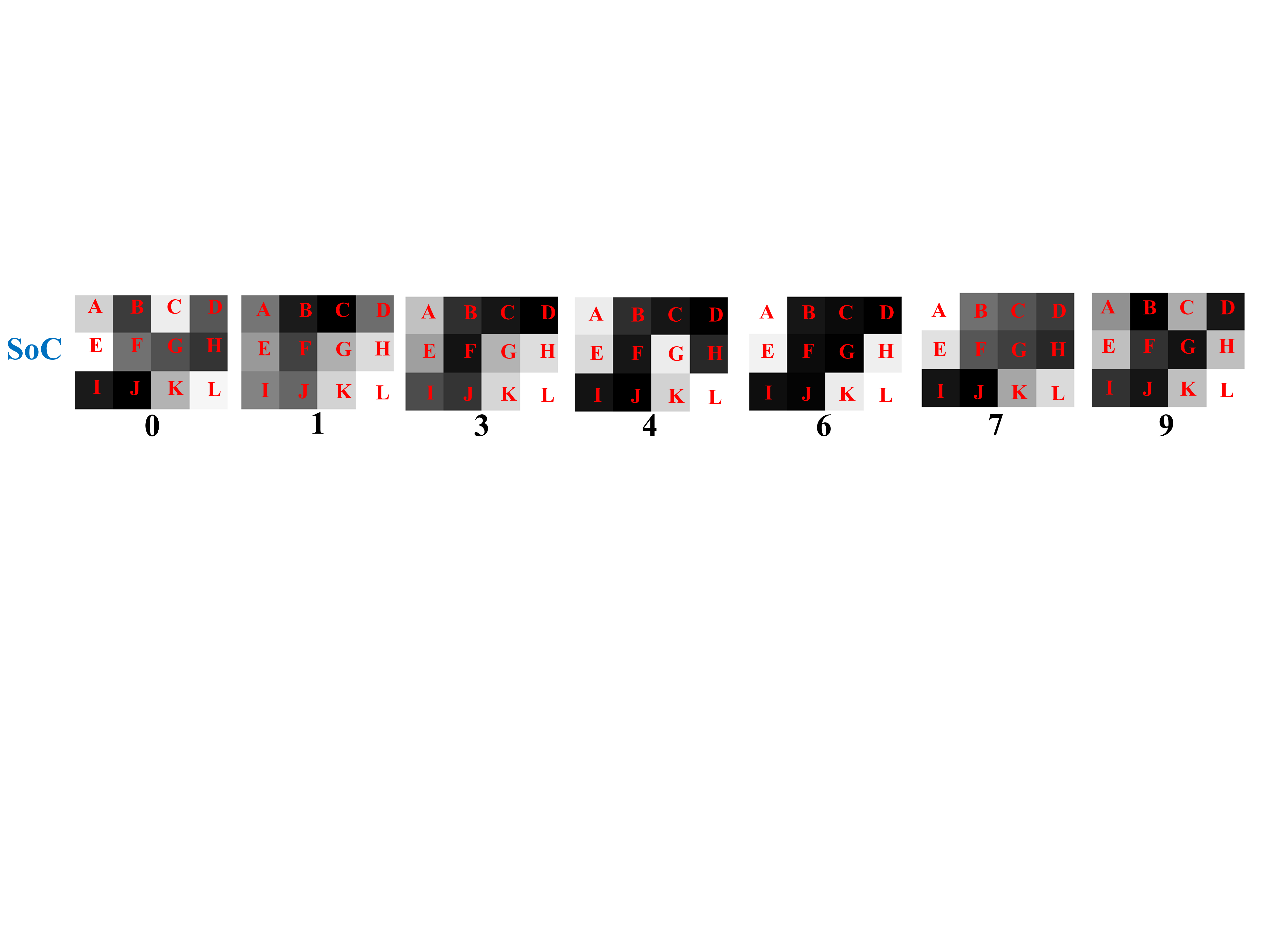
**

**Figure S10**. Grayscale images in SoC for Braille numbers 0, 1, 3, 4, 6, 7, and 9.

**
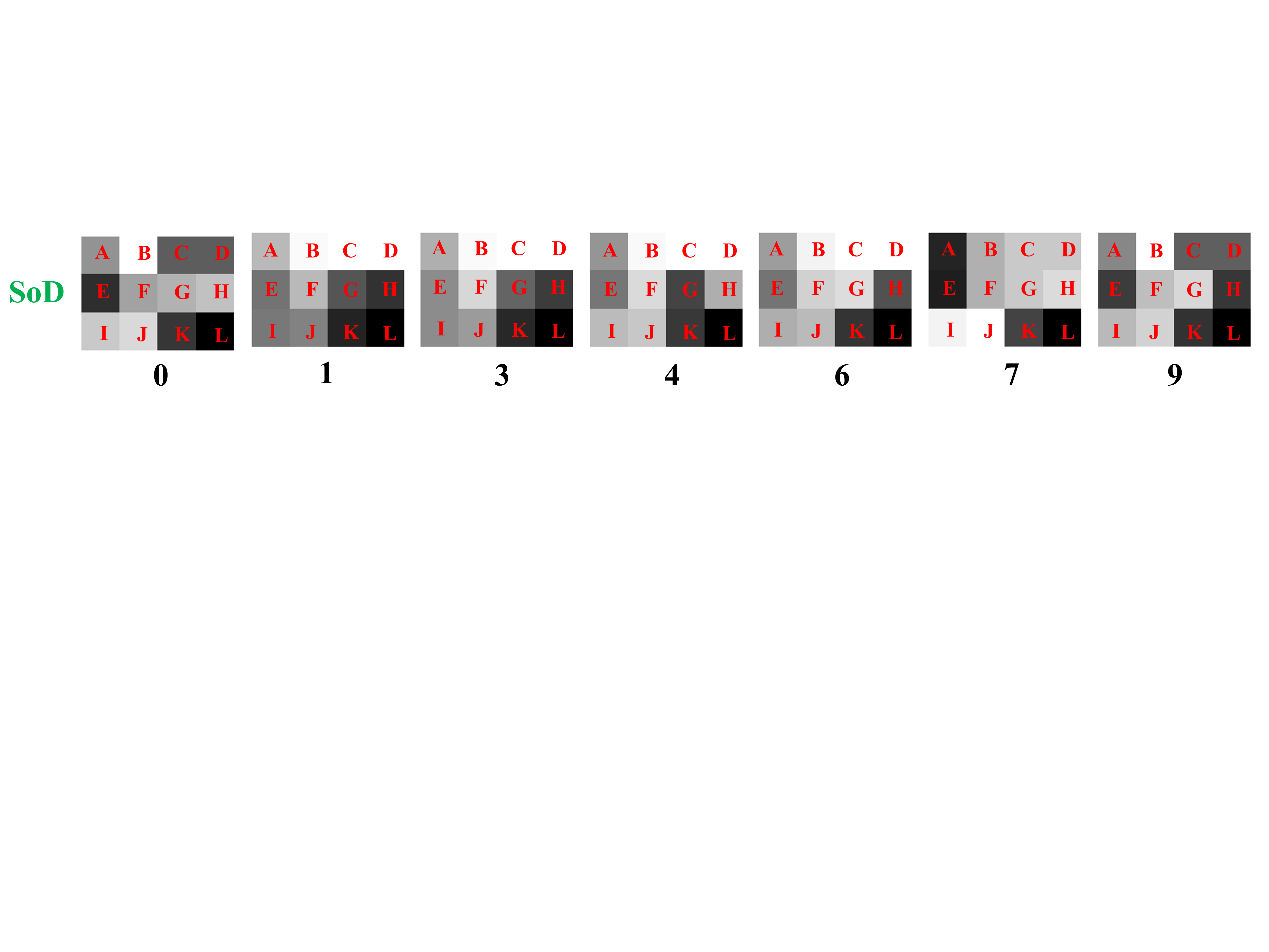
**

**Figure S11**. Grayscale images in SoD for Braille numbers 0, 1, 3, 4, 6, 7, and 9.

**
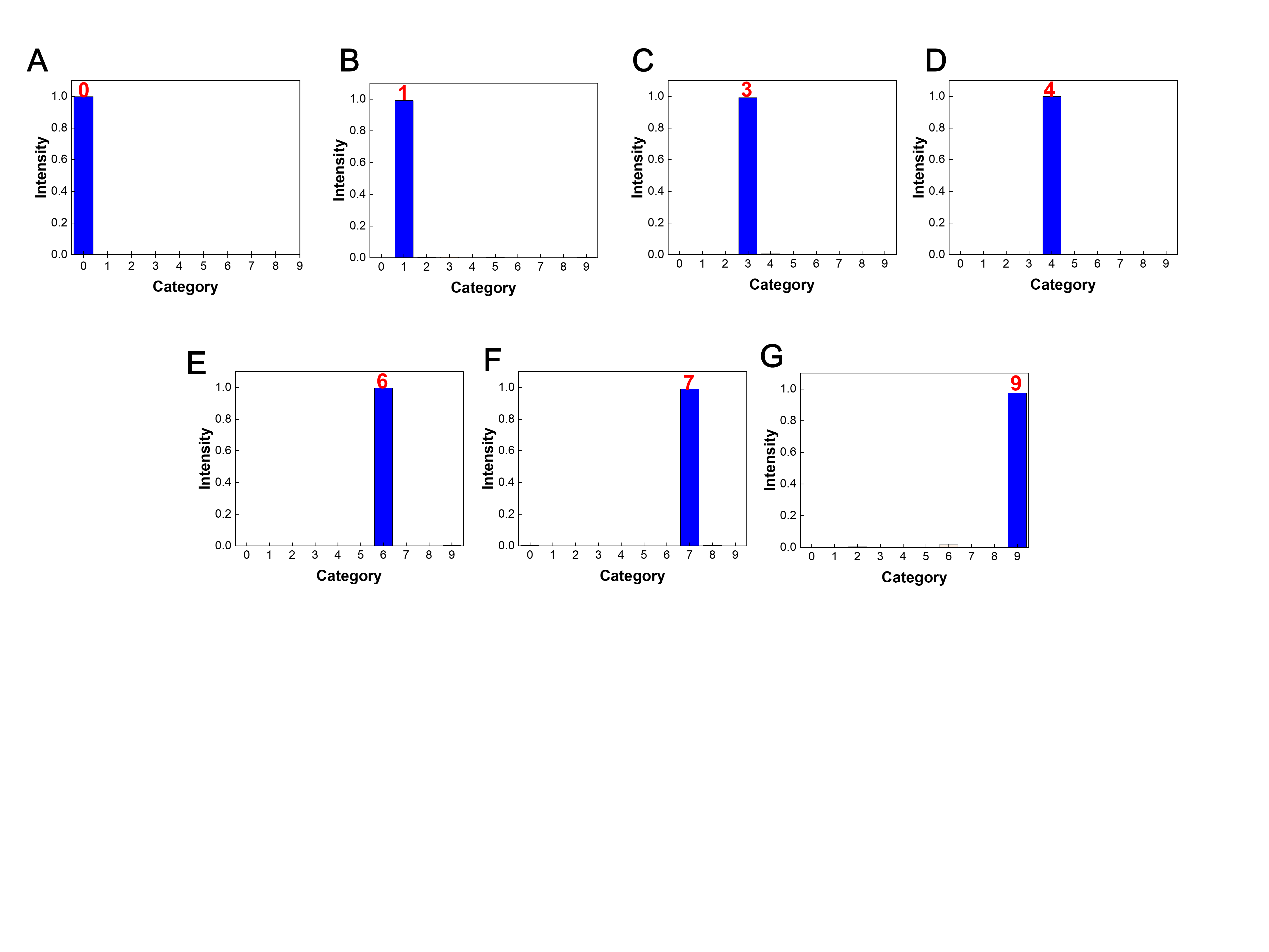
**

**Figure S12**. ANN-based output intensity distributions in SoC for Braille numbers 0, 1, 3, 4, 6, 7, and 9.

**
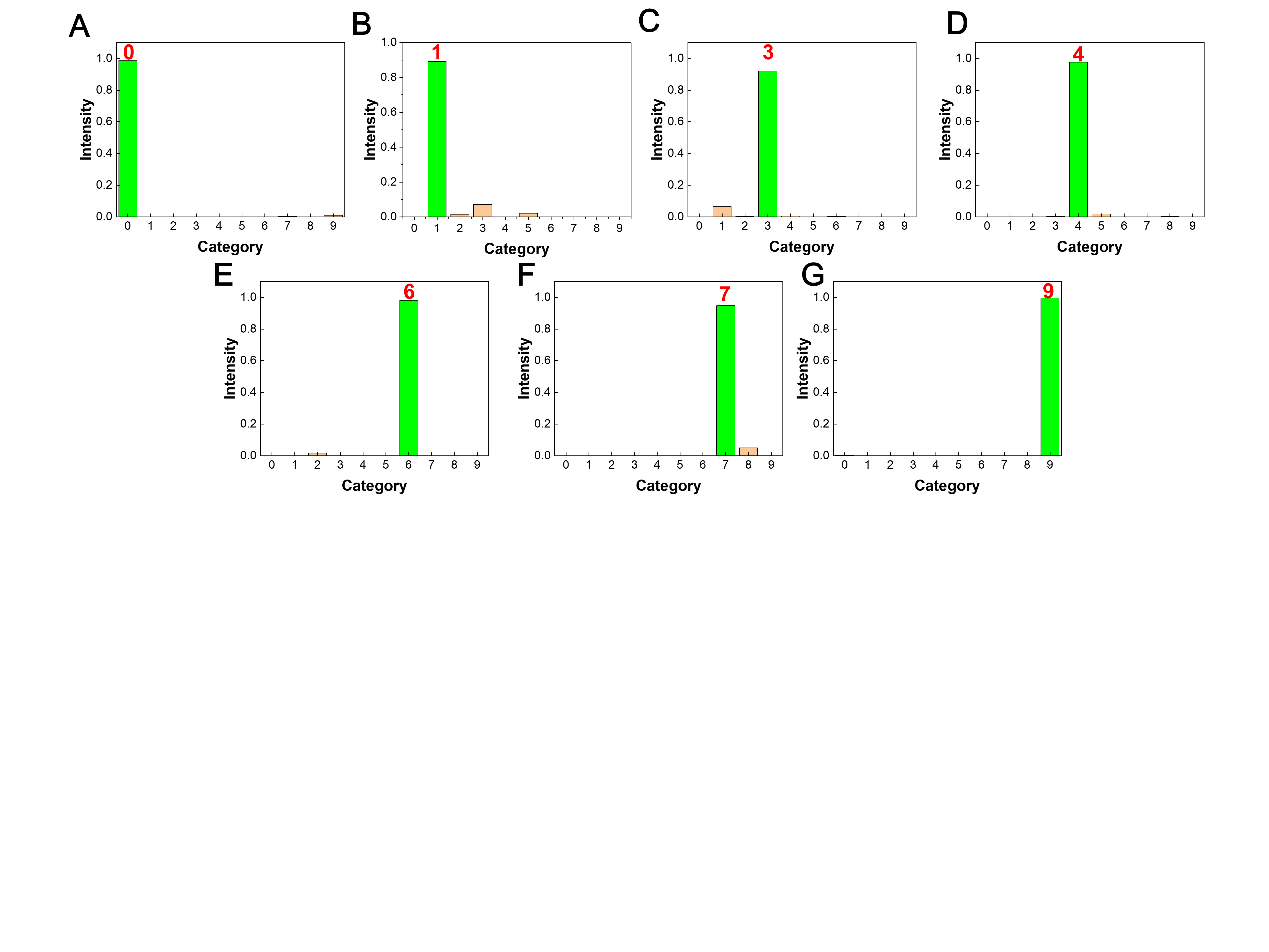
**

**Figure S13**. ANN-based output intensity distributions in SoD for Braille numbers 0, 1, 3, 4, 6, 7, and 9.


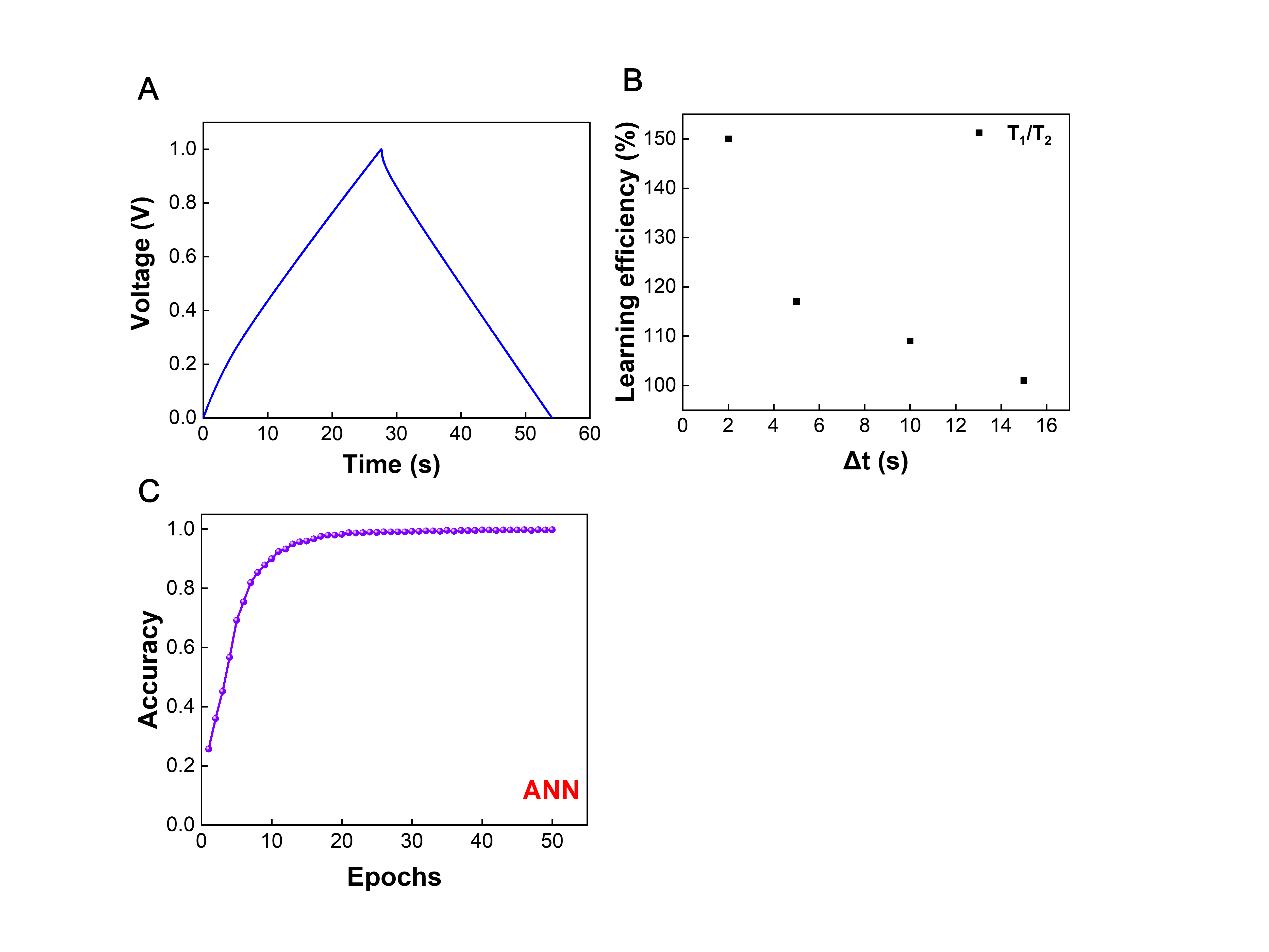


**Figure S14**. A) GCD curves of a graphene supercapacitor at a current density of 0.02 mA/cm^2^. B) Learning efficiency based on graphene supercapacitors. B) Recognition accuracy of graphene-based supercapacitors for Braille in ANN models.

**
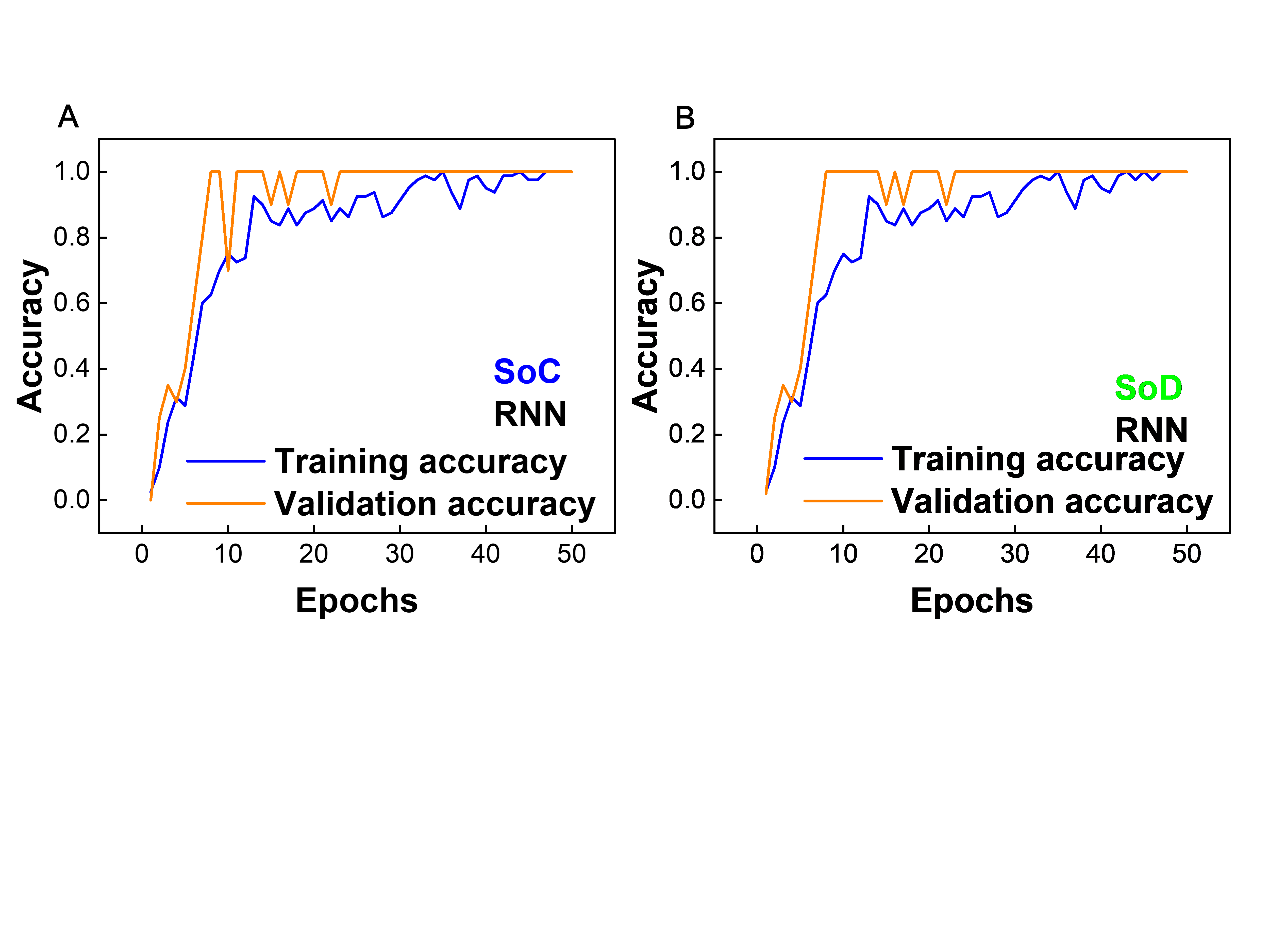
**

**Figure S15**. RNN-based accuracies for recognition of Braille numbers 0-9 in SoC (A) and SoD (B).

**
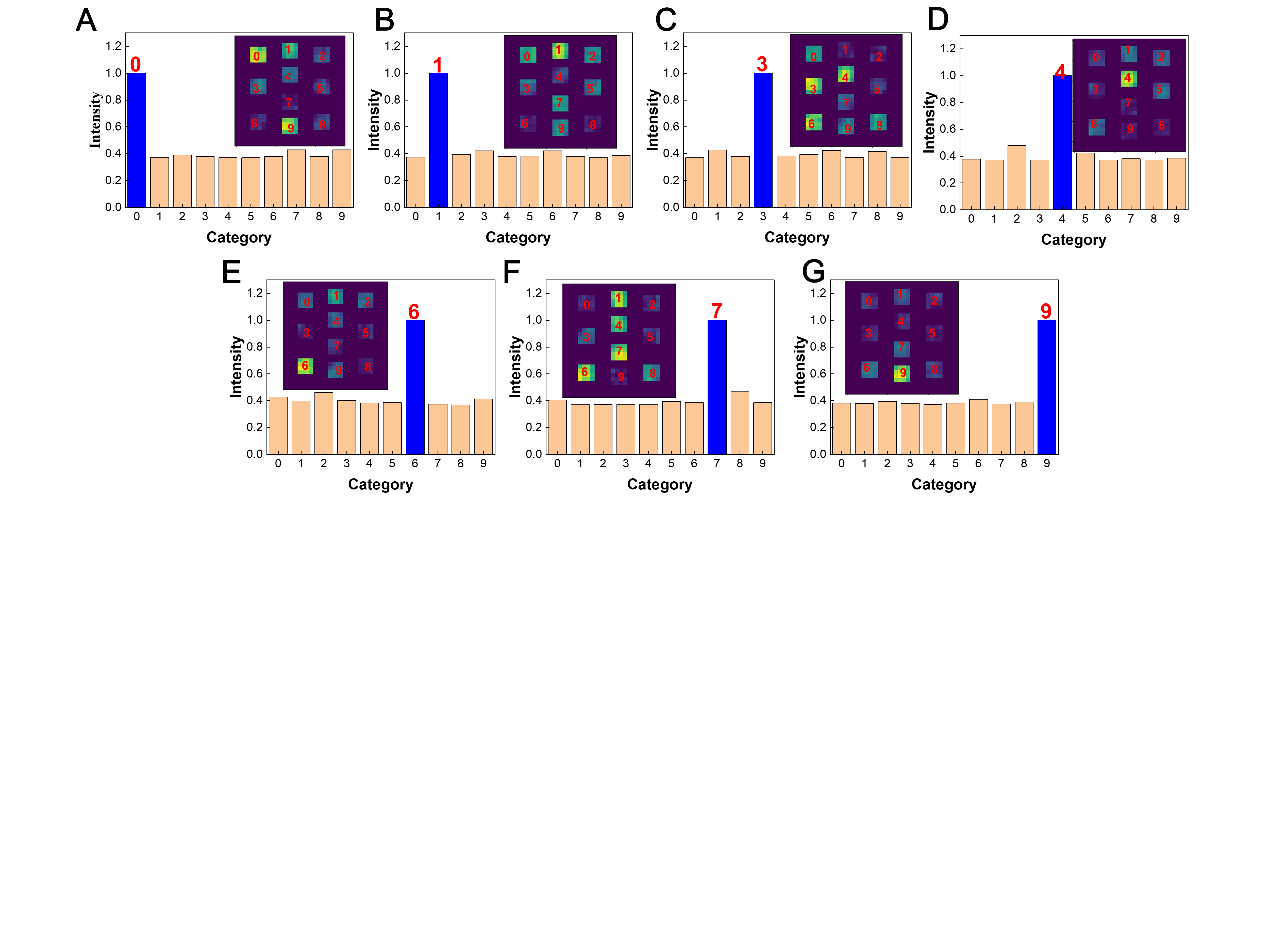
**

**Figure S16**. D^2^NN-based output intensity distributions in SoC for Braille numbers 0, 1, 3, 4, 6, 7, and 9.

**
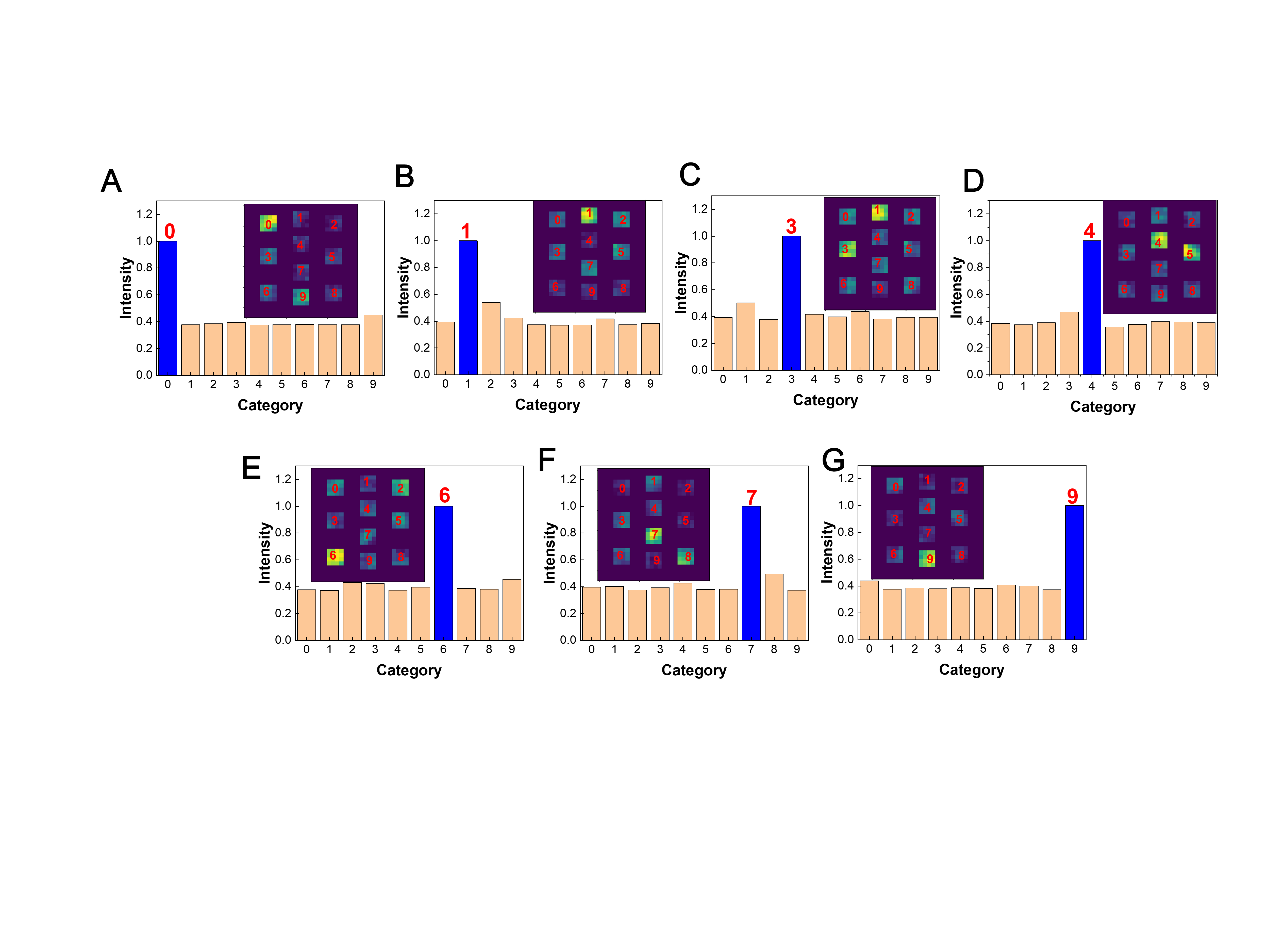
**

**Figure S17**. D^2^NN-based output intensity distributions in SoD for Braille numbers 0, 1, 3, 4, 6, 7, and 9.


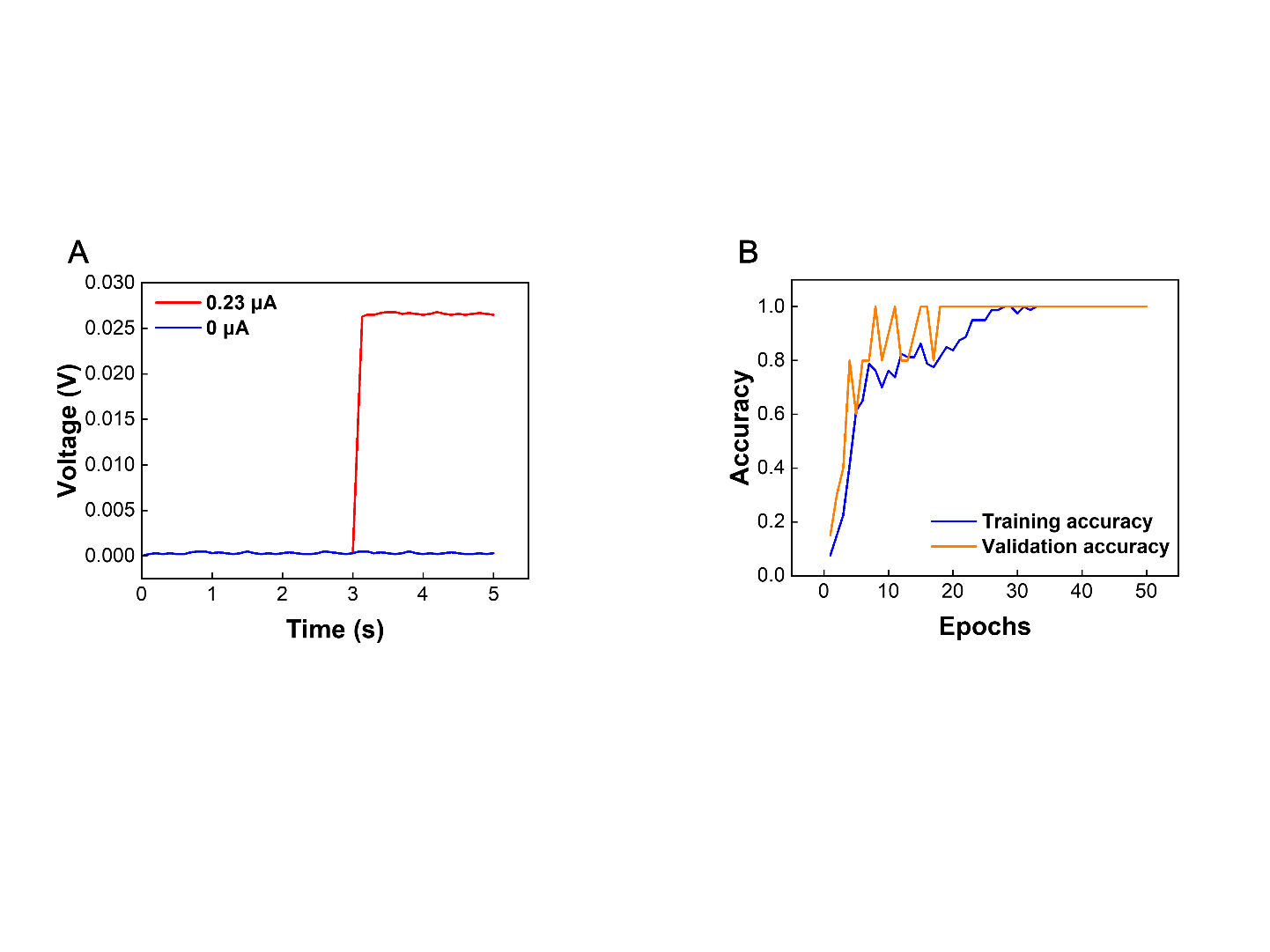


**Figure S18**. A) Voltage response of a Ti₃C₂T_x_ supercapacitor under a 0.13-second current pulse of 0.23 μA. B) RNN-based accuracy for the pulse recognition.
